# Learning the solution operator of parametric partial differential equations with physics-informed DeepONets

**DOI:** 10.1126/sciadv.abi8605

**Published:** 2021-09-29

**Authors:** Sifan Wang, Hanwen Wang, Paris Perdikaris

**Affiliations:** 1 Graduate Group in Applied Mathematics and Computational Science, University of Pennsylvania, Philadelphia, PA 19104, USA.; 2 Department of Mechanical Engineering and Applied Mechanics, University of Pennsylvania, Philadelphia, PA 19104, USA.

## Abstract

Partial differential equations (PDEs) play a central role in the mathematical analysis and modeling of complex dynamic processes across all corners of science and engineering. Their solution often requires laborious analytical or computational tools, associated with a cost that is markedly amplified when different scenarios need to be investigated, for example, corresponding to different initial or boundary conditions, different inputs, etc. In this work, we introduce physics-informed DeepONets, a deep learning framework for learning the solution operator of arbitrary PDEs, even in the absence of any paired input-output training data. We illustrate the effectiveness of the proposed framework in rapidly predicting the solution of various types of parametric PDEs up to three orders of magnitude faster compared to conventional PDE solvers, setting a previously unexplored paradigm for modeling and simulation of nonlinear and nonequilibrium processes in science and engineering.

## Teaser

Enabling the rapid emulation of parametric differential equations with physics-informed deep operator networks.

## INTRODUCTION

As machine learning (ML) methodologies take center stage across diverse disciplines in science and engineering, there is an increased interest in adopting data-driven methods to analyze, emulate, and optimize complex physical systems. The dynamic behavior of such systems is often described by conservation and constitutive laws expressed as systems of partial differential equations (PDEs) (*1*). A classical task then involves the use of analytical or computational tools to solve such equations across a range of scenarios, e.g., different domain geometries, input parameters, and initial and boundary conditions (IBCs). Mathematically speaking, solving these so-called parametric PDE problems involves learning the solution operator that maps variable input entities to the corresponding latent solutions of the underlying PDE system. Tackling this task using traditional tools [e.g., finite element methods (*2*)] bears a formidable cost, as independent simulations need to be performed for every different domain geometry, input parameter, or IBCs. This challenge has motivated a growing literature on reduced-order methods (*3*–*9*) that leverage existing datasets to build fast emulators, often at the price of reduced accuracy, stability, and generalization performance (*10*, *11*). More recently, ML tools are actively developed to infer solutions of PDEs (*12*–*18*); however, most existing tools can only accommodate a fixed given set of input parameters or IBCs. Nevertheless, these approaches have found wide applicability across diverse applications including fluid mechanics (*19*, *20*), heat transfer (*21*, *22*), bioengineering (*23*, *24*), materials (*25*–*28*), and finance (*29*, *30*), showcasing the remarkable effectiveness of ML techniques in learning black box functions, even in high-dimensional contexts (*31*). A natural question then arises: Can ML methods be effective in building fast emulators for solving parametric PDEs?

Solving parametric PDEs requires learning operators (i.e., maps between infinite dimensional function spaces) instead of functions (i.e., maps between finite dimensional vector spaces), thus defining a new and relatively under explored realm for ML-based approaches. Neural operator methods (*32*–*34*) represent the solution map of parametric PDEs as an integral Hilbert-Schmidt operator, whose kernel is parametrized and learned from paired observations, either using local message passing on a graph-based discretization of the physical domain (*32*, *33*) or using global Fourier approximations in the frequency domain (*34*). By construction, neural operators methods are resolution independent (i.e., the model can be queried at any arbitrary input location), but they require large training datasets, while their involved implementation often leads to slow and computationally expensive training loops. More recently, Lu *et al*. (*35*) has presented a novel operator learning architecture coined as DeepONet that is motivated by the universal approximation theorem for operators (*36*, *37*). DeepONets still require large annotated datasets consisting of paired input-output observations, but they provide a simple and intuitive model architecture that is fast to train, while allowing for a continuous representation of the target output functions that is independent of resolution. Beyond deep learning approaches, operator-valued kernel methods (*38*, *39*) have also been demonstrated as a powerful tool for learning nonlinear operators, and they can naturally be generalized to neural networks acting on function spaces (*40*), but their applicability is generally limited due to their computational cost. Here, we should again stress that the aforementioned techniques enable inference in abstract infinite-dimensional Banach spaces (*41*), a paradigm shift from current ML practice that mainly focuses on learning functions instead of operators. Recent theoretical findings also suggest that the sample complexity of deep neural networks (*31*, *42*, *43*), and DeepONets in particular (*44*), can circumvent the curse of dimensionality in certain scenarios.

While the aforementioned methodologies have demonstrated early promise across a range of applications (*45*–*49*), their application to solving parametric PDEs faces two fundamental challenges. First, they require a large corpus of paired input-output observations. In many realistic scenarios, the acquisition of such data involves the repeated evaluation of expensive experiments or costly high-fidelity simulators, thus generating sufficient large training datasets that may be prohibitively expensive. Ideally, one would wish to be able to train such models without any observed data at all (i.e., given only knowledge of the PDE form and its corresponding IBCs). The second challenge relates to the fact that, by construction, the methods outlined above can only return a crude approximation to the target solution operator in the sense that the predicted output functions are not guaranteed to satisfy the underlying PDE. Recent efforts (*16*, *50*–*53*) attempt to address some of these challenges by designing appropriate architectures and loss functions for learning discretized operators (i.e., maps between high-dimensional Euclidean spaces). Although these approaches can relax the requirement for paired input-output training data, they are limited by the resolution of their underlying mesh discretization and, consequently, need modifications to their architecture for different resolutions/discretizations to achieve consistent convergence [if at all possible, as demonstrated in (*32*)].

In this work, we aim to address the aforementioned challenges by exploring a simple yet remarkably effective extension of the DeepONet framework (*35*). Drawing motivation from physics-informed neural networks (*14*), we recognize that the outputs of a DeepONet model are differentiable with respect to their input coordinates, therefore allowing us to use automatic differentiation (*54*, *55*) to formulate an appropriate regularization mechanism for biasing the target output functions to satisfy the underlying PDE constraints. This yields a simple procedure for training physics-informed DeepONet models even in the absence of any training data for the latent output functions, except for the appropriate IBCs of a given PDE system. By constraining the outputs of a DeepONet to approximately satisfy an underlying governing law, we observe substantial improvements in predictive accuracy (up to one to two orders of magnitude reduction in predictive errors), enhanced generalization performance even for out-of-distribution prediction and extrapolation tasks, as well as enhanced data efficiency (up to 100% reduction in the number of examples required to train a DeepONet model). Hence, we demonstrate how physics-informed DeepONet models can be used to solve parametric PDEs without any paired input-output observations, a setting for which existing approaches for operator learning in Banach spaces fall short. Moreover, a trained physics-informed DeepONet model can generate PDE solutions up to three orders of magnitude faster compared to traditional PDE solvers. Together, the computational infrastructure developed in this work can have broad technical impact in reducing computational costs and accelerating scientific modeling of complex nonlinear, nonequilibrium processes across diverse applications including engineering design and control, Earth System science, and computational biology.

## RESULTS

**Fig. 1.**
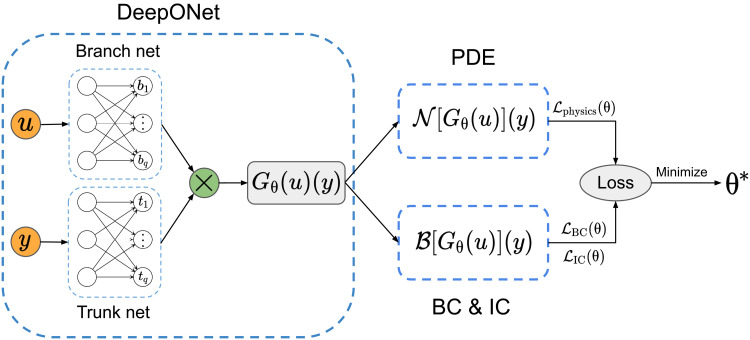
Making DeepONets physics informed. The DeepONet architecture (*35*) consists of two subnetworks, the branch net for extracting latent representations of input functions and the trunk net for extracting latent representations of input coordinates at which the output functions are evaluated. A continuous and differentiable representation of the output functions is then obtained by merging the latent representations extracted by each subnetwork via a dot product. Automatic differentiation can then be used to formulate appropriate regularization mechanisms for biasing the DeepONet outputs to satisfy a given system of PDEs. BC, boundary conditions; IC, initial conditions.

The proposed physics-informed DeepONet architecture is summarized in Fig. 1. Motivated by the universal approximation theorem for operators (*35*, *36*), the architecture features two neural networks coined as the “branch” and “trunk” networks, respectively; the automatic differentiation of which enables us to learn the solution operator of arbitrary PDEs. The associated loss functions, performance metrics, computational cost, hyperparameters, and training details are discussed in the Supplementary Materials. In the following, we demonstrate the effectiveness of physics-informed DeepONets across a series of comprehensive numerical studies for solving various types of parametric PDEs. A summary of the different benchmarks considered is presented in Table 1. It is worth emphasizing that, in all cases, the proposed deep learning models are trained without any paired input-output data, assuming only knowledge of the governing equation and its corresponding initial or boundary conditions.

**Table 1.** Summary of benchmarks for assessing the performance of physics-informed DeepONets across various types of parametric differential equations. The reported test error corresponds to the relative *L*^2^ prediction error of the trained model, averaged over all examples in the test dataset (see eq. S20).

**Governing law**	**Equation form**	**Random input**	**Test error**
Linear ODE	$\frac{\mathrm{ds}(x)}{\mathrm{dx}}=u(x)$	Forcing terms	0.33 ± 0.32%
Diffusion reaction	$\frac{\partial s}{\partial t}=D\frac{\partial^{2}s}{\partial x^{2}}+ks^{2}+u(x)$	Source terms	0.45 ± 0.16%
Burgers’	$\frac{\partial s}{\partial t}+s\frac{\partial s}{\partial x}-\nu \frac{\partial^{2}s}{x^{2}}=0$	Initial conditions	1.38 ± 1.64%
Advection	$\frac{\partial s}{\partial t}+u\frac{\partial s}{\partial x}=0$	Variable coefficients	2.24 ± 0.68%
Eikonal	∥ ∇ *s*∥_2_ = 0	Domain geometries	0.42 ± 0.11%

### Solving a parametric ordinary differential equation

We begin with a pedagogical example involving the antiderivative operator. The underlying governing law corresponds to an initial value problem described by the following ordinary differential equation (ODE)

$$\frac{\mathrm{ds}(x)}{\mathrm{dx}}=u(x),x\in [0,1]$$ (1)

s(0)=0 (2)

**Fig. 2.**
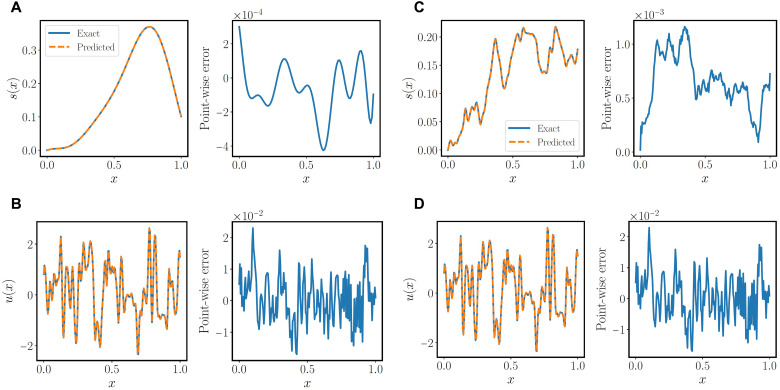
Solving a one-dimensional parametric ODE. (**A** and **B**) Exact solution and residual versus the predictions of a trained physics-informed DeepONet for a representative input function sampled from a GRF with length scale *l* = 0.2. (**C** and **D**) Exact solutions and corresponding ODE residuals versus the predictions of a trained physics-informed DeepONet with Fourier feature embeddings (*56*) for a representative input function sampled from a GRF with length scale *l* = 0.01. The predicted residual *u*(*x*) is computed via automatic differentiation (*55*).

Here, we aim to learn the solution operator mapping any forcing term *u*(*x*) to the ODE solution *s*(*x*) using a physics-informed DeepONet. The model is trained on random realizations of *u*(*x*) generated by sampling a Gaussian random field (GRF) as detailed in the Supplementary Materials, while prediction accuracy is measured in new unseen realizations that are not used during model training. Results for one representative input sample *u*(*x*) from the test dataset are presented in Fig. 2. It is evident that an excellent agreement can be achieved between the physics-informed DeepONet predictions and the ground truth. More impressively, below, we show that physics-informed DeepONets can also accommodate irregular input functions by using an appropriate neural network architecture, such as a Fourier features network (*56*) for their trunk. As shown in Fig. 2, the predicted solutions *s*(*x*) and their corresponding ODE residuals *u*(*x*) obtained by a physics-informed DeepONet with a Fourier feature trunk network are in excellent agreement with the exact solutions for this benchmark. Additional systematic studies and visualizations are provided in the Supplementary Materials (see figs. S1 to S11 and tables S6 to S10). On the basis of these observations, we may also conclude that physics-informed DeepONets can be regarded as a class of deep learning models that greatly enhance and generalize the capabilities of physics-informed neural networks (*57*), which are limited to solving ODEs and PDEs for a given set of input parameters that remain fixed during both the training and prediction phases (see tables S7 and S8 for a more detailed comparison).

It is also worth pointing out that the trained physics-informed DeepONet is even capable of yielding accurate predictions for out-of-distribution test data. To illustrate this, we create a test dataset by sampling input functions from a GRF with a larger length scale of *l* = 0.2 (recall that the training data for this case is generated using *l* = 0.01). The corresponding relative *L*^2^ prediction error averaged over 1000 test examples is measured as 0.7%. Additional visualizations of the model predictions for this out-of-distribution prediction task can be found in the Supplementary Materials (fig. S9).

### Diffusion-reaction dynamics

Our next example involves an implicit operator described by a nonlinear diffusion-reaction PDE with a source term *u*(*x*)

$$\frac{\partial s}{\partial t}=D\frac{\partial^{2}s}{\partial x^{2}}+ks^{2}+u(x),(x,t)\in (0,1]\times (0,1]$$ (3)

assuming zero IBCs, while *D* = 0.01 is the diffusion coefficient and *k* = 0.01 is the reaction rate. Here, we aim to learn the solution operator for mapping source terms *u*(*x*) to the corresponding PDE solutions *s*(*x*). The model is trained on random realizations of *u*(*x*) generated by sampling a GRF as detailed in the Supplementary Materials, while prediction accuracy is measured in new unseen realizations that are not used during model training.

**Fig. 3.**
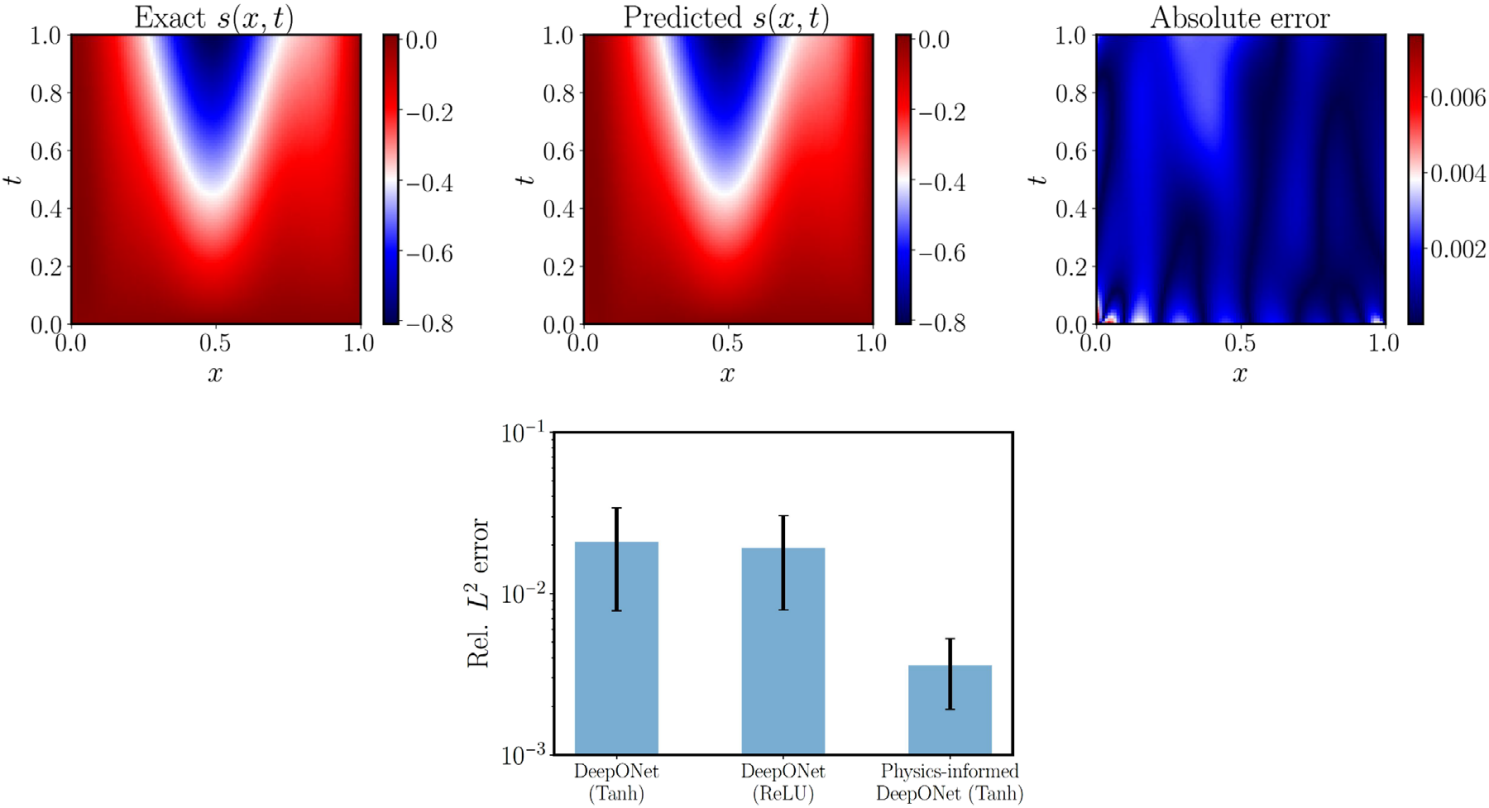
Solving a parametric diffusion-reaction system. (**Top**) Exact solution versus the prediction of a trained physics-informed DeepONet for a representative example in the test dataset. (**Bottom**) Mean and SD of the relative *L*^2^ prediction error of a trained DeepONet (with paired input-output training data) and a physics-informed DeepONet (without paired input-output training data) over 1000 examples in the test dataset. The mean and SD of the relative *L*^2^ prediction are ∼1.92 ± 1.12% (DeepONet) and ∼0.45 ± 0.16% (physics-informed DeepONet), respectively. The physics-informed DeepONet yields ∼80% improvement in prediction accuracy with 100% reduction in the dataset size required for training. Tanh, hyperbolic tangent; ReLU, rectified linear unit.

The top panels of Fig. 3 show the comparison between the predicted and the exact solution for a random test input sample. More visualizations for different input samples can be found in the Supplementary Materials (fig. S12). We observe that the physics-informed DeepONet predictions achieve an excellent agreement with the corresponding reference solutions. Furthermore, we provide a comparison against the conventional DeepONet formulation recently put forth by Lu *et al*. (*35*). This case necessitates observations of paired input-output pairs [*u*(*x*), *s*(*x*, *t*)] to be provided as training data, as no physical constraints are leveraged during model training. The mean and SD of relative *L*^2^ errors of the conventional DeepONet and physics-informed DeepONet over the test dataset are visualized in the bottom panel of Fig. 3. The average relative *L*^2^ error of DeepONet and physics-informed DeepONet are ∼1.92 and ∼0.45%, respectively. In contrast to the conventional DeepONet that is trained on paired input-output measurements, the proposed physics-informed DeepONet can yield much more accurate predictions even without any paired training data (except for the specified IBCs). In our experience, predictive accuracy can be generally improved by using a larger batch size during training. A study of the effect of batch size for training physics-informed DeepONets can be found in the Supplementary Materials (figs. S13 and S16). A series of convergence studies aiming to illustrate how predictive accuracy is affected by the number of input sensor locations *m* and different neural network architectures is also presented in the Supplementary Materials (fig. S14).

### Burgers’ transport dynamics

To highlight the ability of the proposed framework to handle nonlinearity in the governing PDEs, we consider the one-dimensional (1D) Burgers’ benchmark investigated in Li *et al*. (*34*)

$$\frac{\mathrm{ds}}{\mathrm{dt}}+s\frac{\mathrm{ds}}{\mathrm{dx}}-\nu \frac{d^{2}s}{dx^{2}}=0,(x,t)\in (0,1)\times (0,1]$$ (4)

s(x,0)=u(x),x∈(0,1) (5)

with periodic boundary conditions

s(0,t)=s(1,t) (6)

$$\frac{\mathrm{ds}}{\mathrm{dx}}(0,t)=\frac{\mathrm{ds}}{\mathrm{dx}}(1,t)$$ (7)

where *t* ∈ (0,1), the viscosity is set to ν = 0.01, and the initial condition *u*(*x*) is generated from a GRF ∼𝒩(0,25^2^(−Δ + 5^2^*I*)^−4^), satisfying the periodic boundary conditions.

Our goal here is to use the proposed physics-informed DeepONet model to learn the solution operator mapping initial conditions *u*(*x*) to the full spatiotemporal solution *s*(*x*, *t*) of the 1D Burgers’ equation. To this end, the model is trained on random realizations of *u*(*x*) generated by sampling a GRF as detailed in the Supplementary Materials, while prediction accuracy is measured in new unseen realizations that are not used during model training.

**Fig. 4.**
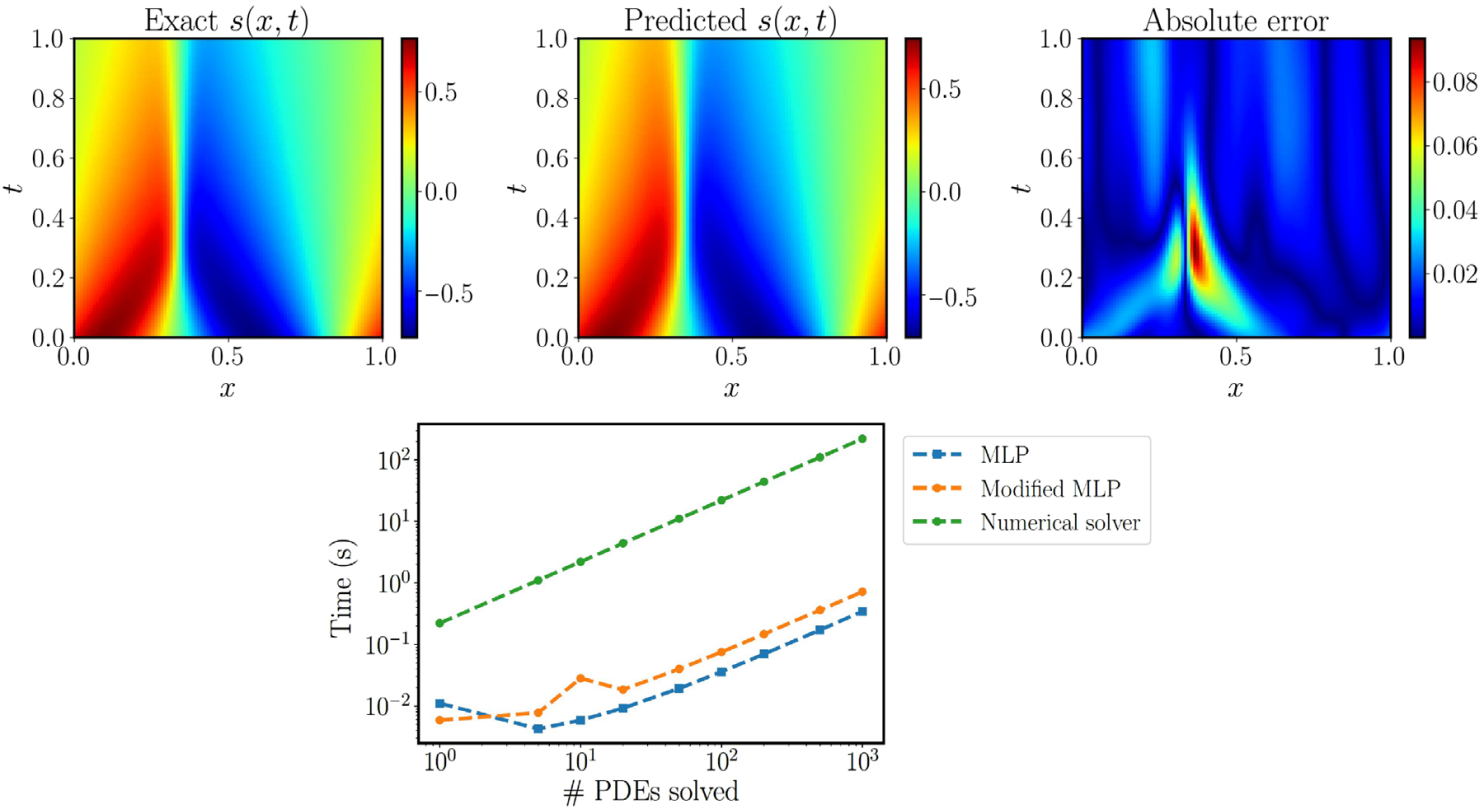
Solving a parametric Burgers’ equation. (**Top**) Exact solution versus the prediction of the best-trained physics-informed DeepONet. The resulting relative *L*^2^ error of the predicted solution is 3%. (**Bottom**) Computational cost (s) for performing inference with a trained physics-informed DeepONet model [conventional or modified multilayer perceptron (MLP) architecture], as well as corresponding timing for solving a PDE with a conventional spectral solver (*58*). Notably, a trained physics-informed DeepONet model can predict the solution of 𝒪(10^3^) time-dependent PDEs in a fraction of a second, up to three orders of magnitude faster compared to a conventional PDE solver. Reported timings are obtained on a single NVIDIA V100 graphics processing unit (GPU).

The average relative *L*^2^ error of the best trained model is ∼1.38% (see figs. S17 to S19). The physics-informed DeepONet achieves the comparable accuracy compared to Fourier operator methods (*34*), albeit the latter has been only tested for a simpler case corresponding to ν = 0.1 and requires training on a large corpus of paired input-output data. Furthermore, visualizations corresponding to the worst example in the test dataset are shown in the top panels of Fig. 4. One can see that the predicted solution achieves a good agreement against the reference solution, with a the relative *L*^2^ error of 3.30%. Here, we must also emphasize that a trained physics-informed DeepONet model can rapidly predict the entire spatiotemporal solution of the Burgers equation in ∼10 ms. Inference with physics-informed DeepONets is trivially parallelizable, allowing for the solution of 𝒪(10^3^) PDEs in a fraction of a second, yielding up to three orders of magnitude in speed up compared to a conventional spectral solver (*58*), see the bottom panel of Fig. 4.

Despite the promising results presented here, we must note the need for further methodological advances toward enhancing the accuracy and robustness of physics-informed DeepONets in tackling PDE systems with stiff, turbulent, or chaotic dynamics. For example, we have observed that the predictive accuracy of physics-informed DeepONets degrades in regions where the PDE solution exhibits steep gradients; a behavior that is pronounced as the viscosity parameter in the Burgers equation is further decreased (see fig. S20 and table S11 for more details and quantitative results). We conjecture that these issues can be tackled in the future by designing of more specialized architectures that are tailored to the dynamic behavior of a given PDE, as well as more effective optimization algorithms for training.

### Advection equation

This example aims to investigate the performance of physics-informed DeepONets for tackling advection-dominated PDEs; a setting for which conventional approaches to reduced-order modeling faces significant challenges (*7*, *10*, *11*). To this end, we consider a linear advection equation with variable coefficients

$$\frac{\partial s}{\partial t}+u(x)\frac{\partial s}{\partial x}=0,(x,t)\in (0,1)\times (0,1)$$ (8)

with the IBC

s(x,0)=f(x) (9)

s(0,t)=g(t) (10)

where *f*(*x*) = sin (π*x*) and $g(t)=\text{sin}\;(\frac{\pi }{2}t)$. To make the input function *u*(*x*) strictly positive, we let $u(x)=v(x)-{\text{min}}_{x}v(x)+1$, where *v*(*x*) is sampled from a GRF with a length scale *l* = 0.2. The goal is to learn the solution operator *G* mapping variable coefficients *u*(*x*) to associated solutions *s*(*x*, *t*) (see the Supplementary Materials for more details).

**Fig. 5.**
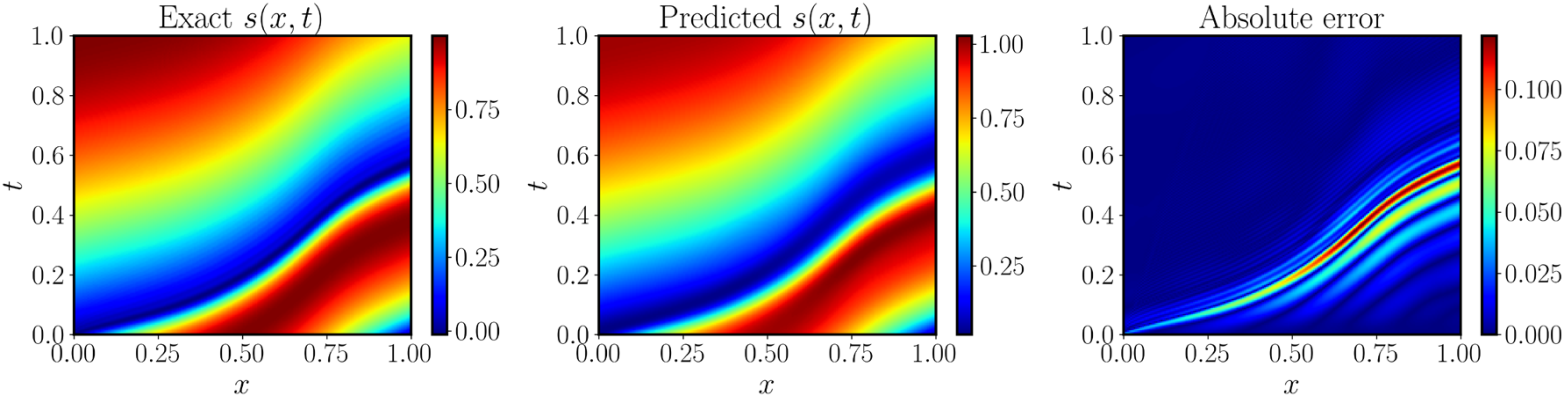
Solving a parametric advection equation. Exact solution versus the prediction of a trained physics-informed DeepONet for a representative example in the test dataset.

As shown in Fig. 5, the trained physics-informed DeepONet is able to achieve an overall good agreement with the reference PDE solution, although some inaccuracies can be observed in regions where the solution exhibits steep gradients (similarly to the Burgers’ example discussed above; see additional visualizations presented in fig. S21). The resulting relative *L*^2^ prediction averaged over all examples in the test dataset is 2.24%, leading to the conclusion that physics-informed DeepONets can be effective surrogates for advection-dominated PDEs.

### Eikonal equation

Our last example aims to highlight the capability of the proposed physics-informed DeepONet to handle different types of input functions. To this end, let us consider a 2D eikonal equation of the form

$$\begin{array}{c} ∥\nabla s(x){∥}_{2}=1\\ s(x)=0,x\in \partial \Omega \end{array}$$ (11)

where **x** = (*x*, *y*) ∈ ℝ^2^ denotes 2D spatial coordinates, and Ω is an open domain with a piece-wise smooth boundary ∂Ω. A solution to the above equation is a signed distance function measuring the distance of a point in Ω to the closest point on the boundary ∂Ω, i.e.

$$s(x)=\{\begin{array}{cc} d(x,\partial \Omega ) & \text{if}\;x\in \Omega \\ -d(x,\partial \Omega ) & \text{if}\;x\in \Omega^{c} \end{array}$$

where *d*( · , · ) is a distance function defined as

$$d(x,\partial \Omega )≔\underset{y\in \partial \Omega }{\text{inf}}d(x,y)$$ (12)

Signed distance functions (SDFs) have recently sparked increased interest in the computer vision and graphics communities as a tool for shape representation learning (*59*). This is because SDFs can continuously represent abstract shapes or surfaces implicitly as their zero-level set, yielding high-quality shape representations, interpolation, and completion from partial and noisy input data (*59*). In this example, we seek to learn the solution map from a well-behaved closed curve Γ to its associated signed distance function, i.e., the solution of the eikonal equation defined in Eq. 11. As a benchmark we consider different airfoil geometries from the University of Illinois--Urbana-Champaign (UIUC) database (*60*), a subset of which is used to train the model (see the Supplementary Materials for more details).

**Fig. 6.**
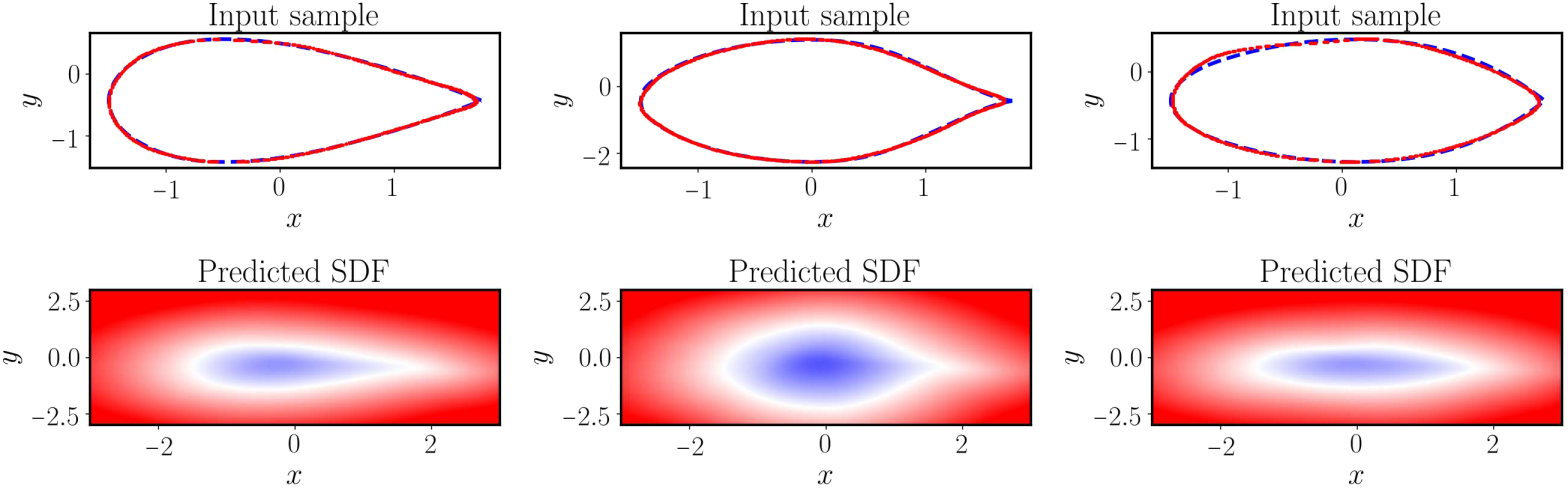
Solving a parametric eikonal equation (airfoils). (**Top**) Exact airfoil geometry versus the zero-level set obtained from the predicted signed distance function for three different input examples in the test dataset. (**Bottom**) Predicted signed distance function of a trained physics-informed DeepONet for three different airfoil geometries in the test dataset.

The trained DeepONet model is then capable of predicting the solution of the eikonal equation for any given input airfoil geometry. To evaluate its performance, we visualize the zero-level set of the learned signed distance function and compare it with the exact airfoil geometry. As shown in Fig. 6, the zero-level sets achieve a good agreement with the exact airfoil geometries. One may conclude that the proposed framework is capable of achieving an accurate approximation of the exact signed distance function. Additional systematic studies and quantitative comparisons are provided in the Supplementary Materials (see figs. S23 to S25).

## DISCUSSION

This paper presents physics-informed DeepONets, a novel deep learning framework for approximating nonlinear operators in infinite-dimensional Banach spaces. Leveraging automatic differentiation, we present a simple yet remarkably effective mechanism for biasing the outputs of DeepONets toward physically consistent predictions, allowing us to realize significant improvements in predictive accuracy, generalization performance, and data efficiency compared to existing operator learning techniques. An even more intriguing finding is that physics-informed DeepONets can learn the solution operator of parametric ODEs and PDEs, even in the absence of any paired input-output training data. This capability is introducing a new radical way of simulating nonlinear and nonequilibrium phenomena across different applications in science and engineering up to three orders of magnitude faster compared to conventional solvers.

Given the prominent role that PDEs play in the mathematical analysis, modeling, and simulation of complex physical systems, the physics-informed DeepONet architecture can be broadly applied in science and engineering since PDEs are prevalent across diverse problem domains including fluid mechanics, electromagnetics, quantum mechanics, and elasticity. However, despite the early promise demonstrated here, numerous technical questions remain open and require further investigation. Motivated by the successful application of Fourier feature networks (*56*), it is natural to ask the following: For a given parametric governing law, what is the optimal features embedding or network architecture of a physics-informed DeepONet? Recently, Wang *et al*. (*61*) proposed a multiscale Fourier feature network to tackle PDEs with multiscale behavior. Such an architecture may be potentially used as the backbone of physics-informed DeepONets to learn multiscale operators and solve multiscale parametric PDEs. Another question arises from the possibility of achieving improved performance by assigning weights in the physics-informed DeepONet loss function. It has been shown that these weights play an important role in enhancing the trainability of constrained neural networks (*62*–*64*). Therefore, it is natural to ask the following: What are the appropriate weights to use for training physics-informed DeepONets? How to design effective algorithms for accelerating training and ensuring accuracy and robustness in the predicted outputs? We believe that addressing these questions will not only enhance the performance of physics-informed DeepONets but also introduce a paradigm shift in how we model and simulate complex, nonlinear, and multiscale physical systems across diverse applications in science and engineering.

## METHODS

DeepONets (*35*) present a specialized deep learning architecture that encapsulates the universal approximation theorem for operators (*36*). Here, we illustrate how DeepONets can be effectively applied to learning the solution operator of parametric PDEs. Here, the terminology “parametric PDEs” refers to the fact that some parameters of a given PDE system are allowed to vary over a certain range. These input parameters may include, but are not limited to, the shape of the physical domain, the initial or boundary conditions, constant or variable coefficients (e.g., diffusion or reaction rates), source terms, etc. To describe such problems in their full generality, let (𝒰, 𝒱, 𝒮) be a triplet of Banach spaces and 𝒩 : 𝒰 × 𝒮 → 𝒱 be a linear or nonlinear differential operator. We consider general parametric PDEs taking the form

N(u,s)=0 (13)

where **u** ∈ 𝒰 denotes the parameters (i.e., input functions) and **s** ∈ 𝒮 denotes the corresponding unknown solutions of the PDE system. Specifically, we assume that, for any **u** ∈ 𝒰, there exists an unique solution **s** = **s**(**u**) ∈ 𝒮 to 13 (subject to appropriate IBCs). Then, we can define the solution operator *G* : 𝒰 → 𝒮 as

G(u)=s(u) (14)

Following the original formulation of Lu *et al.* (*35*), we represent the solution map *G* by an unstacked DeepONet *G*_θ_, where θ denotes all trainable parameters of the DeepONet network. As illustrated in Fig. 1, the unstacked DeepONet is composed of two separate neural networks referred to as the branch and trunk networks, respectively. The branch network takes **u** as input and returns a features embedding [*b*_1_, *b*_2_, …, *b_q_*]*^T^* ∈ ℝ*^q^* as output, where **u** = [**u**(**x**_1_), **u**(**x**_2_), …, **u**(**x***_m_*)] represents a function **u** ∈ 𝒰 evaluated at a collection of fixed locations ${\left\{x_{i}\right\}}_{i=1}^{m}$. The trunk network takes the continuous coordinates **y** as inputs and outputs a features embedding [*t*_1_, *t*_2_, …, *t_q_*]*^T^* ∈ *ℝ^q^*. To obtain the final output of the DeepONet, the outputs of the branch and trunk networks are merged together via a dot product. More specifically, a DeepONet *G*_θ_ prediction of a function **u** evaluated at **y** can be expressed by

$$G_{\theta }(u)(y)=\sum_{k=1}^{q}\underset{\text{branch}}{\underbrace{b_{k}(u(x_{1}),u(x_{2}),\ldots ,u(x_{m}))}}\underset{\text{trunk}}{\underbrace{t_{k}(y)}}$$ (15)

where θ denotes the collection of all trainable weight and bias parameters in the branch and trunk networks.

Notice that the outputs of a DeepONet model are continuously differentiable with respect to their input coordinates. Therefore, one may use automatic differentiation (*54*, *55*) to formulate an appropriate regularization mechanism for biasing the target output functions to satisfy any given differential constraints.

Consequently, we may then construct a “physics-informed” DeepONet by formulating the following loss function

$$L(\theta )=L_{\text{operator}}(\theta )+L_{\text{physics}}(\theta )$$ (16)

where

$$L_{\text{operator}}(\theta )=\frac{1}{\mathrm{NP}}\sum_{i=1}^{N}\sum_{j=1}^{P}{|G_{\theta }(u^{\left(i\right)})(y_{u,j}^{\left(i\right)})-G(u^{\left(i\right)})(y_{u,j}^{\left(i\right)})|}^{2}$$ (17)

$$L_{\text{physics}}(\theta )=\frac{1}{\mathrm{NQm}}\sum_{i=1}^{N}\sum_{j=1}^{Q}\sum_{k=1}^{m}{|N(u^{\left(i\right)}(x_{k}),G_{\theta }(u^{\left(i\right)})(y_{r,j}^{\left(i\right)}))|}^{2}$$ (18)

Here, ${\left\{u^{\left(i\right)}\right\}}_{i=1}^{N}$ denotes *N* separate input functions sampled from 𝒰. For each **u**^(*i*)^, ${\left\{y_{u,j}^{\left(i\right)}\right\}}_{j=1}^{P}$ are *P* locations that are determined by the data observations, initial or boundary conditions, etc. Besides, ${\left\{y_{r,j}^{\left(i\right)}\right\}}_{j=1}^{Q}$ is a set of collocation points that can be randomly sampled in the domain of *G*(**u**^(*i*)^). As a consequence, ℒ_operator_(θ) fits the available solution measurements while ℒ_physics_(θ) enforces the underlying PDE constraints. Contrary to the fixed sensor locations of ${\left\{x_{i}\right\}}_{i=1}^{m}$, we remark that the locations of ${\left\{y_{u,j}^{\left(i\right)}\right\}}_{j=1}^{P}$ and ${\left\{y_{r,j}^{\left(i\right)}\right\}}_{j=1}^{Q}$ may vary for different *i*, thus allowing us to construct a continuous representation of the output functions **s** ∈ 𝒮. More details on how this general framework can be adapted the different PDE systems presented in Results—including the choice of neural network architectures, formulation of loss functions, and training details—are provided in the Supplementary Materials.

## Supplementary Materials

### This PDF file includes:

Supplementary Materials and Methods Supplementary Text Tables S1 to S11 Figs. S1 to S25 References
