# Supplementary material for: Learning the solution operator of parametric partial differential equations with physics-informed DeepONets
Source: Sci Adv. 2021 Sep 29;7(40):eabi8605. doi: 10.1126/sciadv.abi8605 (PMC8480920; doi:10.1126/sciadv.abi8605)
Supplement: Supplementary file 1 — Supplementary Materials and Methods Supplementary Text Tables S1 to S11 Figs. S1 to S25 References [file sciadv.abi8605_sm.pdf]

Science Advances
AAAS

Supplementary Materials for

Learning the solution operator of parametric partial differential equations with physics-informed DeepONets

Sifan Wang, Hanwen Wang, Paris Perdikaris*

*Corresponding author. Email: pgp@seas.upenn.edu

Published 29 September 2021, *Sci. Adv.* **7**, eabi8605 (2021)
DOI: 10.1126/sciadv.abi8605

This PDF file includes:

Supplementary Materials and Methods
Supplementary Text
Tables S1 to S11
Figs. S1 to S25
References

Materials and Methods

Nomenclature

Table S1 summarizes the main symbols and notation used in this work.

Notation	Description
$\mathbf{u}(\cdot)$	an input function
$\mathbf{s}(\cdot)$	a solution to a parametric PDE
G	an operator
G_θ	an unstacked DeepONet representation of the operator G
θ	all trainable parameters of a DeepONet
$\{\mathbf{x}_i\}_{i=1}^m$	m sensor points where input functions $\mathbf{u}(\mathbf{x})$ are evaluated
$[u(\mathbf{x}_1), u(\mathbf{x}_2), \ldots, u(\mathbf{x}_m)]$	an input of the branch net, representing the input function u
$\mathbf{y}$	an input of the trunk net, a point in the domain of $G(u)$
N	number of input samples in the training data-set
M	number of locations for evaluating the input functions u
P	number of locations for evaluating the output functions $G(u)$
Q	number of collocation points for evaluating the PDE residual
GRF	a Gaussian random field
SDF	a signed distance function
l	length scale of a Gaussian random field
$\mathcal{L}_{\text{operator}}(\theta)$	a loss function to fit available observations
$\mathcal{L}_{\text{physics}}(\theta)$	a loss function to fit the underlying physical laws

Table S1: *Nomenclature*: Summary of the main symbols and notation used in this work.

Data generation

In most examples, we model random input functions $\mathbf{u}(\mathbf{x})$ using mean-zero Gaussian random fields (GRF) (*68*) as

$$\mathbf{u}(\mathbf{x}) \sim \mathcal{GP}\left(0, k_l\left(\mathbf{x}_1, \mathbf{x}_2\right)\right),$$

with an exponential quadratic covariance kernel $k_l\left(x_1, x_2\right) = \exp\left(-\left\|x_1 - x_2\right\|^2 / 2l^2\right)$ with a length scale parameter $l > 0$. The parameter l will be used to control the complexity of the sampled input functions, and in general larger $l > 0$ leads to smoother $\mathbf{u}$.

In general, a DeepONet training data-set is a triplet $[\mathbf{u}, \mathbf{y}, G(\mathbf{u})(\mathbf{y})]$ with following structure

$$[\mathbf{u}, \mathbf{y}, G(\mathbf{u})(\mathbf{y})] = \left[\begin{bmatrix} \vdots \\ \mathbf{u}^{(i)}(\mathbf{x}_1), \mathbf{u}^{(i)}(\mathbf{x}_2), \cdots, \mathbf{u}^{(i)}(\mathbf{x}_m) \\ \mathbf{u}^{(i)}(\mathbf{x}_1), \mathbf{u}^{(i)}(\mathbf{x}_2), \cdots, \mathbf{u}^{(i)}(\mathbf{x}_m) \\ \vdots \\ \mathbf{u}^{(i)}(\mathbf{x}_1), \mathbf{u}^{(i)}(\mathbf{x}_2), \cdots, \mathbf{u}^{(i)}(\mathbf{x}_m) \\ \vdots \end{bmatrix}, \begin{bmatrix} \vdots \\ \mathbf{y}_1^{(i)} \\ \mathbf{y}_2^{(i)} \\ \vdots \\ \mathbf{y}_P^{(i)} \\ \vdots \end{bmatrix}, \begin{bmatrix} \vdots \\ G(\mathbf{u}^{(i)})(\mathbf{y}_1^{(i)}) \\ G(\mathbf{u}^{(i)})(\mathbf{y}_2^{(i)}) \\ \vdots \\ G(\mathbf{u}^{(i)})(\mathbf{y}_P^{(i)}) \\ \vdots \end{bmatrix}\right]. \tag{19}$$

It is important to highlight that each input function $\mathbf{u}^{(i)} = [\mathbf{u}^{(i)}(\mathbf{x}_1), \mathbf{u}^{(i)}(\mathbf{x}_2), \cdots, \mathbf{u}^{(i)}(\mathbf{x}_m)]$ repeats itself for P times. In other words, suppose that $\mathbf{u} \in \mathcal{U}, G(\mathbf{u}) \in \mathcal{V}$ are scalar-valued functions, $\{\mathbf{u}^{(i)}\}_{i=1}^N$ are N sample functions, and, for each sample $\mathbf{u}^{(i)}$, $G(\mathbf{u}^{(i)})$ is evaluated at P different locations $\{\mathbf{y}^{(i)}\}_{j=1}^P \subset \mathbb{R}^d$, then the tensor dimensions constituting a DeepONet training data-set $\mathbf{u}, \mathbf{y}, G(\mathbf{u})(\mathbf{y})$ are $(N \times P, m), (N \times P, d), (N \times P, 1)$ respectively.

Hyper-parameter settings

In all examples considered in this work, the branch net and the trunk net are equipped with hyperbolic tangent activation functions (Tanh), except for the Eikonal benchmark (airfoils), where ELU activations were employed. Physics-informed DeepONet models are trained via mini-batch gradient descent with a batch-size of 10,000 using the Adam optimizer (*69*) with

default settings. In this work, we tuned these hyper-parameters manually, without attempting to find the absolute best hyper-parameter setting. This process can be automated in the future leveraging effective techniques for meta-learning and hyper-parameter optimization (*70*).

Case	Input functions	m	#u Train	P	Q	# u Test	Iterations
Anti-derivative	$l = 0.2$	100	10^4	1	100	1,000	4×10^4
ODE (regular)	$l = 0.2$	100	10^4	1	100	1,000	4×10^4
ODE (irregular)	$l = 0.01$	200	10^4	1	200	1,000	3×10^5
Diffusion-reaction	$l = 0.2$	100	10^4	100	100	1,000	1.2×10^5
Burgers'	–	100	10^3	100	2,500	1,000	2×10^5
Advection	$l = 0.2$	100	10^3	200	2,500	1,000	3×10^5
Eikonal (circles)	–	100	10^3	100	1,000	1,000	8×10^4
Eikonal (airfoils)	–	250	10^3	250	1,000	500	1.2×10^5

Table S2: Default hyper-parameter settings for each benchmark employed in this work (unless otherwise stated).

Case	Trunk width	Trunk depth	Branch width	Branch depth
Anti-derivative operator	50	5	50	5
1D ODE (regular input)	50	5	50	5
1D ODE (irregular input)	200	5	200	5
Diffusion-reaction	50	5	50	5
Advection	100	7	100	7
Burger	100	7	100	7
Eikonal (circles)	50	6	50	6
Eikonal (airfoils)	100	7	100	7

Table S3: Physics-informed DeepONet architectures for each benchmark employed in this work (unless otherwise stated).

Case	Trunk width	Trunk depth	Branch width	Branch depth
Antiderivative operator	100	3	100	3
1D ODE (regular input)	50	5	50	5
1D ODE (irregular input)	50	5	50	5
Diffusion-reaction	50	5	50	5

Table S4: Conventional DeepONet (*35*) architectures for each corresponding benchmark (unless otherwise stated).

Performance metrics

The error metric employed throughout all numerical experiments to assess model performance is the relative L^2 norm. Specifically, the reported test errors correspond to the mean of the relative L^2 error of a trained physics-informed DeepONet model over all examples in the test data-set, i.e

$$\text{Test error} := \frac{1}{N}\sum_{i=1}^{N}\frac{\|G_\theta(u^{(i)})(y) - G(u^{(i)})(y)\|_2}{\|G(u^{(i)})(y)\|_2}, \tag{20}$$

where N denotes the number of examples in the test data-set and y is typically a set of equi-spaced points in the domain of $G(u)$. Here $G_\theta(u^{(i)})(y)$ denotes the predicted DeepONet outputs, while $G(u^{(i)})(y)$ corresponds to the ground truth target functions.

Computational cost

Training: Table S5 summarizes the computational cost (hours) of training DeepONet and physics-informed DeepONet models with different network architectures. The size of different models as well as network architectures are listed table S4 and S3, respectively. All networks are trained using a single NVIDIA V100 GPU. It can be observed that training a physics-informed DeepONet model is generally slower than training a conventional DeepONet. This is expected as physics-informed DeepONets require to compute the PDE residual via automatic differentiation, yielding a lager computational graph, and, therefore, a higher computational cost.

Case	Model (Architecture)	Training time (hours)
Anti-derivative	DeepONet	0.03
	Physics-informed DeepONet	0.15
ODE (regular)	DeepONet	0.03
	Physics-informed DeepONet	0.15
ODE (irregular)	Physics-informed DeepONet (MLP)	1.61
	Physics-informed DeepONet (FF)	1.37
Diffusion-reaction	DeepONet	1.13
	Physics-informed DeepONet	2.27
Burgers'	Physics-informed DeepONet (MLP)	7.61
	Physics-informed DeepONet (Modified MLP)	9.25
Advection	Physics-informed DeepONet	7.31
Eikonal (circles)	Physics-informed DeepONet	0.76
Eikonal (airfoils)	Physics-informed DeepONet	0.38

Table S5: Computational cost (hours) for training DeepONet and physics-informed DeepONet models across the different becnhmarks and architectures employed in this work. Reported timings are obtained on a single NVIDIA V100 GPU.

Inference: A trained physics-informed DeepONet model can rapidly predict the entire spatio-temporal solution of the Burgers equation in $\sim$10ms. Inference with DeepONets is trivially parallelizable, allowing for the solution of $\mathcal{O}(10^3)$ PDEs in a fraction of a second, yielding up to three orders of magnitude in speed up compared to a traditional spectral solver (*58*) (see Figure 4 in the manuscript).

Supplementary Text

A comparison against DeepONets: Learning anti-derivative operator

In this section, we demonstrate the motivation of the proposed physics-informed DeepONet. Although DeepONets (*35*) and their variants (e.g. DeepM&Mnets (*45*)) have demonstrated great potential in approximating operators and solving multi-physics and multi-scale problems, it is worth pointing out that the learned operator may not be consistent with the underlying physical laws that generated the observed data (e.g., due to the finite capacity of neural networks or lack of sufficient training data). To this end, let us consider a pedagogical example involving a simple initial value problem

$$\frac{ds(x)}{dx} = u(x), \quad x \in [0, 1], \tag{21}$$

with an initial condition $s(0) = 0$. Here, our goal is to learn the anti-derivative operator

$$G : u(x) \longrightarrow s(x) = s(0) + \int_0^x u(t)dt, \quad x \in [0, 1]. \tag{22}$$

To generate a training data-set, we randomly sample 10,000 different functions u from a zero-mean Gaussian process prior with an exponential quadratic kernel using a length scale of $l = 0.2$ (*68*). We also obtain the corresponding 10,000 ODE solutions s by integrating the ODE 21 using an explicit Runge-Kutta method (RK45) (*71*). For each observed pair of (u, s), we choose $m = 100$ sensors $\{x_i\}_{i=1}^m$ uniformly distributed on the time interval $[0, 1]$ and randomly select $P = 1$ observations of $s(\cdot)$ in $[0, 1]$. To generate the test data-set, we repeat the same procedure with $m = 100$ and $P = 100$. The final test data-set contains $1,000$ different samples of random input functions u.

We represent the operator G using the unstacked DeepONet G_θ where both the branch net and the trunk net are two-layer fully-connected neural networks with 100 neurons per hidden layer. Each network is equipped with ReLU activation functions. The network parameters can

be trained by minimizing the following loss

$$\mathcal{L}(\theta) = \frac{1}{N} \sum_{i=1}^{N} \left| G_\theta(\mathbf{u}^{(i)})(y^{(i)}) - s^{(i)}(y^{(i)}) \right|^2, \tag{23}$$

where $\mathbf{u}^{(i)} = [u^{(i)}(x_1), u^{(i)}(x_2), \ldots, u^{(i)}(x_m)]$ represent the input function, and $s^{(i)}(y^{(i)})$ denotes the associated solution of equation 21 evaluated at $y^{(i)}$.

We train the DeepONet model by minimizing the above loss function via gradient descent using the Adam optimizer (*69*) for $40,000$ iterations. Note that the final output of DeepONet is a function of input coordinates $\mathbf{x}$. Thus, we can compute the residual $\frac{ds(x)}{dx}$ of the inferred solution $s(x)$ using automatic differentiation (*55*), and compare the computed residual with $u(x)$ at the sensors $\{x_i\}_{i=1}^m$. Figure S1 shows the comparison of the predicted $s(x)$ and $\frac{ds(x)}{dx}$ against the ground truth for one representative random sample from the test data-set. We can observe a good agreement between the predicted and the exact solution $s(x)$ when using ReLU activation functions. However, the predicted residual $\frac{ds(x)}{dx}$ seems to approximate $u(x)$ with step functions, which leads to a large approximation error. One may postulate that this is due to the non-smoothness of ReLU activations. However, as shown in the same Figure S1, similar poor predictions of both $u(x)$ and $s(x)$ are obtained by repeating the same process using a DeepONet equipped with tanh activations, under exactly the same hyper-parameter settings. Thus, despite the guarantee of universal approximation theorem for operators (*36*), it is possible that DeepONet models may not appropriately learn the correct solution operator in the sense that the predicted output functions are not compatible with the ground truth operator that generated the training data.

Physics-informed neural networks (PINNs) (*14*) can seamlessly integrate the data measurements and physical governing laws by penalizing residuals of partial differential equations in the loss function of a neural network using automatic differentiation (*55*). Motivated by PINNs and our findings in the previous section, we propose a novel model class referred to as "physics-

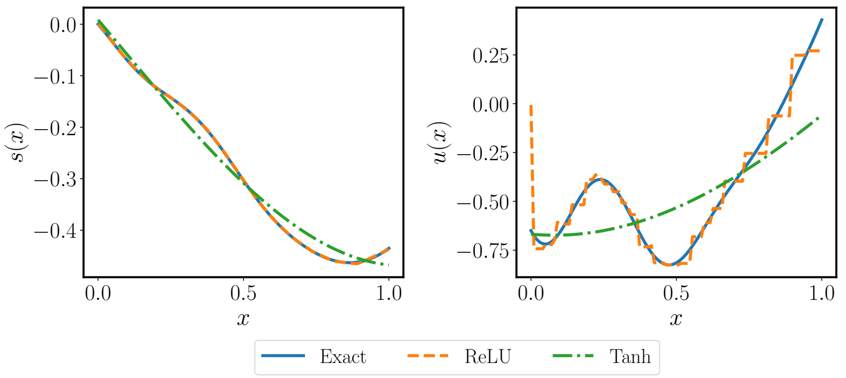

Figure S1: *Learning the anti-derivative operator:* Predicted solution $s(x)$ and residual $u(x)$ versus the ground truth for a representative input function. The results are obtained by training a conventional DeepONet model (*35*) equipped with different activation functions after 40,000 iterations of gradient descent using the Adam optimizer.

informed" DeepONets that enables the DeepONet output functions to be consistent with physical constraints via minimizing the residual of the underlying governing laws in the same manner as PINNs. Specifically, we consider minimizing the following composite loss function

$$\mathcal{L}(\theta) = \mathcal{L}_{\text{operator}}(\theta) + \mathcal{L}_{\text{physics}}(\theta), \tag{24}$$

where $\mathcal{L}_{\text{operator}}(\theta)$ is exactly the same as the loss 23 and

$$\mathcal{L}_{\text{physics}}(\theta) = \frac{1}{Nm}\sum_{i=1}^{N}\sum_{j=1}^{m}\left|\frac{dG_\theta(\mathbf{u}^{(i)})(y)}{dy}\Big|_{y=x_j} - u^{(i)}(x_j)\right|^2. \tag{25}$$

Figure S2 presents the predicted $s(x)$ and $\frac{ds(x)}{dx}$ for the same random sample (see Figure S1) by minimizing the loss function 24 for 40,000 iterations of gradient descent using the Adam optimizer. Evidently, both predictions achieve an excellent agreement with the corresponding reference solutions. This can be further verified by the mean of relative L^2 error of the model predictions reported table S6, from which we may conclude that the physics-informed DeepONet not only attains comparable accuracy to the original DeepONet, but also satisfies the underlying ODE constraint. Another crucial finding is that physics-informed DeepONets are data-efficient and therefore effective in small data regime. To illustrate this, we train both

a DeepONet and a physics-informed DeepONet for different number of training data points (i.e, different number of samples u) and report the mean of the relative L^2 error of $s(x)$ over 1,000 realizations from the test data-set in Figure S3. We observe that conventional DeepONets require more than 10x training data to achieve the same accuracy as their physics-informed counterpart.

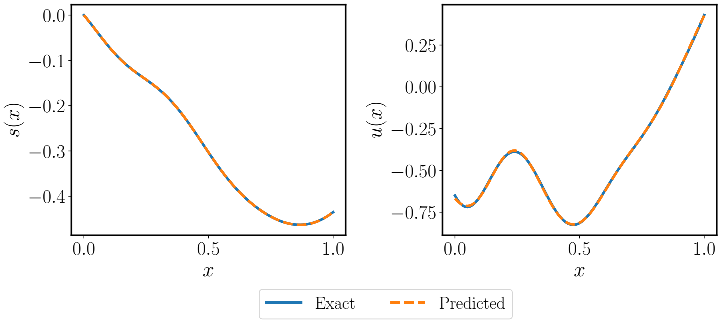

Figure S2: *Learning anti-derivative operator:* Exact solution and residual versus the predictions of a trained physics-informed DeepONet for the same input function as in Figure S1.

Model \ Relative L^2 error	Relative L^2 error of s	Relative L^2 error of u
DeepONet (ReLU)	$5.16e-03 \pm 4.58e-03$	$1.39e-01 \pm 5.58e-02$
DeepONet (Tanh)	$1.89e-01 \pm 1.51e-01$	$6.14e-01 \pm 2.36e-01$
Physics-informed DeepONet (Tanh)	$2.49e-03 \pm 2.74e-03$	$6.29e-03 \pm 3.65e-03$

Table S6: *Learning anti-derivative operator:* Mean and standard deviation of relative L^2 prediction errors of DeepONet and physics-informed DeepONet equipped with ReLU or Tanh activations over 1,000 examples in the test data-set.

A comparison between physics-informed neural networks and DeepONets

Here we would like to discuss the fundamental differences between physics-informed neural networks (*14*) and the proposed physics-informed DeepONets. The goal of physics-informed neural networks (PINNs) is to parametrize and learn the solution of a single partial differential

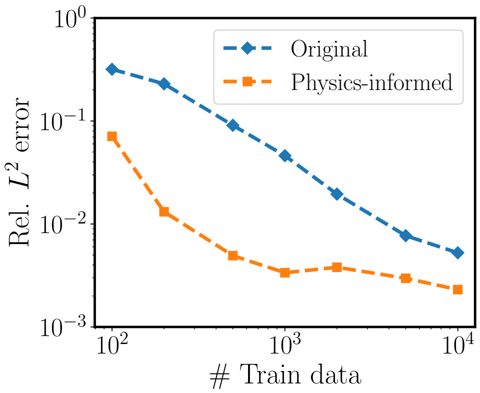

Figure S3: *Learning anti-derivative operator:* Mean of the relative L^2 prediction error of the original DeepONet (*35*) and the physics-informed DeepONet as a function of the number of u samples.

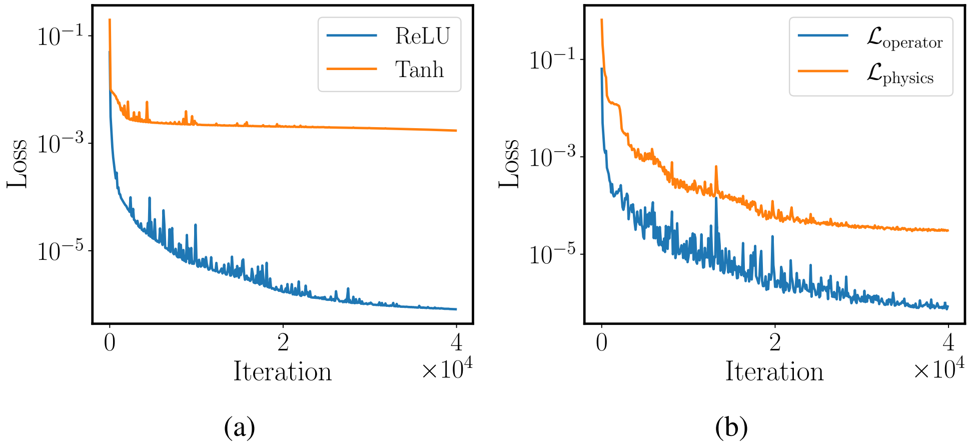

Figure S4: *Learning an anti-derivative operator:* (a) Training loss convergence of a conventional DeepONet model equipped with different activation functions for 40,000 iterations of gradient descent using the Adam optimizer. (b) Training loss convergence of a physics-informed DeepONet equipped with Tanh activations for 40,000 iterations of gradient descent using the Adam optimizer.

equation (PDE), for the case where the PDE parameters are given and remain fixed during model training. These parameters include, but are not limited to, boundary/initial conditions, coefficients, source terms, etc. Consequently, a trained PINNs model can yield an approximated solution a given PDE system with specified parameters, but cannot generalize to other input parameters (at least not unless the model is re-trained). That being said, we should remark that

some transfer learning techniques may be applied in this setting to accelerate the re-training process and enable a PINNs model to learn the associated solutions corresponding to other input parameters, with a reduced computational cost (see (*72*), for example).

In contrast to PINNs, the proposed physics-informed DeepONets aim to parametrize the PDE solution operator that maps different input parameters to the associated PDE solutions. In this setting, one needs to specify a distribution over functions or the function space associated with any variable input parameters before training. As a consequence, from a practical standpoint, perhaps the most important distinction between PINNs and physics-informed DeepONets is that the latter can quickly and accurately infer PDE solutions corresponding to different input parameters through simple model evaluations and without requiring any re-training. It worth pointing out that physics-informed DeepONet can be also applied to solve a single PDE if we fix the input of the branch network in DeepONet architecture. As such, physics-informed DeepONets can be regarded as a class of deep learning models that greatly enhance and generalize the capabilities of PINNs.

To illustrate the above points, here we provide a simple example in which PINNs and physics-informed DeepONets can be compared in equal footing. Specifically, one of the benchmarks considered in the main manuscript corresponding to the following ODE system

$$\frac{ds(x)}{dx} = u(x), \quad x \in [0, 1], \tag{26}$$

$$s(0) = 0, \tag{27}$$

where $u(x)$ is generated by sampling a Gaussian Random Field (GRF) with a length scale $l = 0.2$. Since a standard PINNs model cannot tackle parametric equations, for each forcing term $u(x)$, we employ a separate physics-informed neural network to solve the associated ODE system. Here, the solution is represented by a 5-layer full-connected neural network with 50 neurons per hidden layer and tanh activations. We sample $N = 100$ input functions and train

Model	Rel. L^2 error	Training time (hours)
Physics-informed neural network	$0.07\% \pm 0.08\%$	2.53
Physics-informed DeepONet	$0.33\% \pm 0.32\%$	0.15

Table S7: *Linear ODE:* Relative L^2 prediction error of PINNs and physics-informed DeepONets averaged over all examples in the test data-set.

each physics-informed neural network for $40,000$ iterations using the Adam optimizer. A comparison of PINNs and physics-informed DeepONets is summarized in Table S7. One can see that the computational cost of training PINNs is much greater than training a physics-informed DeepONet because we need to train PINNs for $N = 100$ times. Moreover, for this simple example, PINNs can achieve better predictive accuracy, since each PINNs model is trained to learn the solution of just a single ODE case. On the other hand, physics-informed DeepONets are called to solve the much harder task of learning the ODE solution operator, in which thousands of ODE cases are concurrently considered during model training. From our experience so far, we can empirically claim that if one can be successful in employing PINNs to solve a given ODE/PDE system, then one should be able to use physics-informed DeepONets to solve the parametric version of the same PDE with reasonable accuracy. More interestingly, in follow-up work (*73*), we have shown that physics-informed DeepONets can be employed to solve even more complex problems involving long-time integration for which conventional PINNs approaches fail consistently.

Another question worth asking is: Can we modify the vanilla PINN architecture to solve parametric PDEs? The answer is yes, and the formulation is very simple. Indeed, one can concatenate the input coordinates and the parameters before passing them through a deep neural network, and formulate a physics-informed loss in the same manner. Taking the previous benchmark as an example, we can represent the solution operator $G(u)$ by a fully-connected neural network $s_\theta(x, \mathbf{u})$ where x denotes the input coordinates and $\mathbf{u} = [u(x_1), \dots, u(x_m)]$ denotes a

forcing term evaluated at a set of equi-spaced points $\{x_i\}_{i=1}^m$ in $[0,1]$. Then, a parameterized PINNs model can be trained by minimizing the following the loss function

$$\mathcal{L}(\theta) = \mathcal{L}_{\text{IC}}(\theta) + \mathcal{L}_{\text{physics}}(\theta) \tag{28}$$

$$= \frac{1}{N}\sum_{i=1}^{N}\left|s_\theta(0, \mathbf{u}^{(i)})\right|^2 + \frac{1}{NQ}\sum_{i=1}^{N}\sum_{j=1}^{Q}\left|\frac{ds_\theta(x, \mathbf{u}^{(i)})(x)}{dx}\Big|_{x=x_j} - u^{(i)}(x_j)\right|^2, \tag{29}$$

where we use the same notation as in physics-informed DeepONets.

To investigate the performance of parameterized PINNs and compare them against the proposed physics-informed DeepONets, we performed a series of numerical studies for solving different parameteric PDEs under exactly the same hyper-parameter settings (# iterations, optimizer, learning rate, activations, etc.) The resulting test errors of the trained models are summarized in table S8. One can observe that the test errors of parameterized PINNs are noticeably worse than the physics-informed DeepONet, especially for Eikonal equation benchmark. We believe that the remarkable results of physics-informed DeepONet benefit from the theoretical justification of operator approximation theorems (*35, 36, 44*), as well as the dot product operation in the DeepONet architecture that merges the branch and trunk network outputs, which potentially enhances their expressivity and trainability. Moreover, we expect that DeepONet architecture is less prone to the "curse of dimensionality" as discussed in (*44*), allowing one to accommodate higher-dimensional representations of input functions (i.e. a larger number of input sensor locations m). On the other hand, conventional parameterized PINNs require one to sample collocation points in the joint space of input parameters and domain coordinates, leading to difficulties in training as the dimensionality of the inputs is increased.

Case	Model	Test error
ODE	Physics-informed DeepONet	$0.33\% \pm 0.32\%$
	Parameterized PINN	$5.25\% \pm 5.72\%$
Diffusion-reaction	Physics-informed DeepONet	$0.25\% \pm 0.12\%$
	Parameterized PINN	$1.06\% \pm 0.74\%$
Eikonal equation (circles)	Physics-informed DeepONet	$0.42\% \pm 0.11\%$
	Parameterized PINN	$27.45\% \pm 24.12\%$

Table S8: Relative L^2 prediction error of parameterized PINNs and physics-informed DeepONets across various parametric PDE benchmarks, averaged over all examples in the test dataset. All models are trained under exactly the same hyper-parameter setting (# iterations, optimizer, learning rate, activations, etc.).

Linear ODE system

Recall that the one-dimensional ODE system is described by

$$\frac{ds(x)}{dx} = u(x), \quad x \in [0,1], \tag{30}$$

$$s(0) = 0. \tag{31}$$

Our goal is to learn the solution operator from $u(x)$ to the solution $s(x)$ using physics-informed DeepONets. We represent the operator by a DeepONet G_θ where both branch net and trunk net are 5-layer fully-connected neural networks with 50 neurons per hidden layer and equipped with tanh activations. The corresponding loss function is expressed as

$$\mathcal{L}(\theta) = \mathcal{L}_{\text{operator}}(\theta) + \mathcal{L}_{\text{physics}}(\theta) \tag{32}$$

$$= \frac{1}{N}\sum_{i=1}^{N}\left|G_\theta(\mathbf{u}^{(i)})(0)\right|^2 + \frac{1}{NQ}\sum_{i=1}^{N}\sum_{j=1}^{Q}\left|\frac{dG_\theta(\mathbf{u}^{(i)})(y)}{dy}\Big|_{y=x_j} - u^{(i)}(x_j)\right|^2. \tag{33}$$

Regular input functions

Here, $\mathbf{u}^{(i)} = [u^{(i)}(x_1), u^{(i)}(x_2), \dots, u^{(i)}(x_m)]$, and we sample $N = 10,000$ input functions $u(x)$ from a GRF with length scale $l = 0.2$. Moreover, we take $Q = m = 100$ and $\{x_j\}_{j=1}^{Q}$ are equi-

spaced grid points in $[0, 1]$. From the expression of the loss function, it is worth emphasizing that all "training data" comes the measurements of $u(x)$, and the zero initial condition on $s(0)$ (i.e. no other observations of $s(x)$ are available). Some visualizations of the trained physics-informed DeepONet are presneted in Figure S5.

Moreover, we investigate the performance of the original DeepONet (*35*) in solving this parametric ODE example. To this end, we train a DeepONet by minimizing the loss function $\mathcal{L}_{\text{operator}}(\theta)$ under exactly the same hyper-parameter setting. Representative predicted solutions $s(x)$ for different input samples u are shown in Figure S6. We observe that the conventional DeepONet learns a degenerate map that can fit the initial condition $s(0) = 0$, but returns erroneous predictions for all $x > 0$. These observations can be further quantified in Table S9, which reports the mean and standard deviation of the relative L^2 prediction error for the output functions s and their corresponding ODE residual u over 1,000 examples in the test data-set. Remarkably, the proposed physics-informed DeepONet is trained in the absence of any paired input-output data, but still obtains comparable accuracy to the results shown in Table S9, where the model is trained with a large amount of paired input-output observations.

Irregular input functions

Furthermore, we show that physics-informed DeepONets can accommodate extremely irregular input functions by using appropriate trunk network architectures. To illustrate this, we consider a GRF with a length scale $l = 0.01$ as a prior on the input function space. We take $Q = m = 200$ and repeat the same data generation procedure as before. In this example, the training data-set contains $N = 10,000$ different u samples, while the test data-set contains 1,000 realizations.

Given that the input functions are sampled from a GRF with a relatively small length scale, the associated solutions are expected to exhibit high frequencies. Therefore, we represent the latent operator by a DeepONet with Fourier feature embeddings (*56*), which are able to learn

high-frequency components more effectively. Generally, A random Fourier mapping γ is defined as

$$\gamma(\mathbf{v}) = \begin{bmatrix} \cos(\mathbf{B}\mathbf{v}) \\ \sin(\mathbf{B}\mathbf{v}) \end{bmatrix}, \tag{34}$$

where each entry in $\mathbf{B} \in \mathbb{R}^{m \times d}$ is sampled from a Gaussian distribution $\mathcal{N}(0, \sigma^2)$ and $\sigma > 0$ is a user-specified hyper-parameter. Then, a Fourier feature network (*56*) can be simply constructed using a random Fourier features mapping γ as a coordinate embedding of the inputs, followed by a conventional fully-connected neural network.

In particular, we encode the input functions by a branch net that is a 5-layer fully-connected neural network with 200 neurons per hidden layer. In addition, we apply a Fourier feature embedding (*56*) initialized with $\sigma = 50$ to the input coordinates $\mathbf{y}$ before passing the embedded inputs through a trunk network with the same architecture as the branch net. Some visualizations of the trained model are shown in Figure S7. The results of training the same physics-informed DeepONet without Fourier feature embeddings are presented in Figure S8. One may observe that using a conventional fully-connected trunk networks cannot accurately capture the high-frequency oscillations, leading to a large prediction error. These observations can be further quantified in Table S10, which summarizes the mean and standard derivation of the relative L^2 prediction error of trained physics-informed DeepONets constructed with different network architectures. Although here we have illustrated that an appropriate network architecture plays a prominent role in the performance of DeepONets, a comprehensive investigation of DeepONet architectures is beyond the scope of the present study and will be investigated in future work.

Finally, we describe the details of out-of-distribution prediction of the trained physics-informed DeepONet with Fourier feature networks. We create a test data-set by sampling input functions from a GRF with a larger length-scale of $l = 0.2$ (recall that the training data for this case is generated using $l = 0.01$). The corresponding relative L^2 prediction error averaged over

$1,000$ test examples is measured as 0.7%. Some visualizations of the model predictions for this out-of-distribution prediction task are shown in the Figure S9.

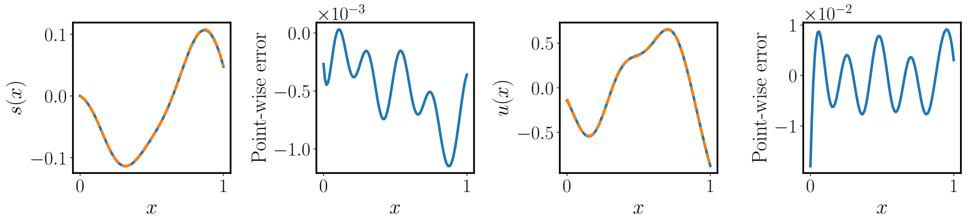

(a)

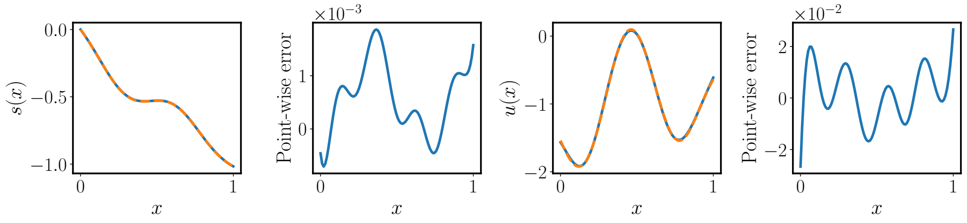

(b)

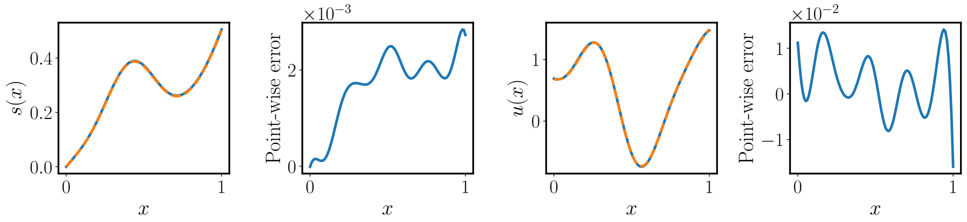

(c)

Figure S5: *Solving a 1D parametric ODE:* Predicted solutions $s(x)$ and corresponding ODE residuals $u(x)$ for a trained physics-informed DeepONet, across three different examples in the test data-set.

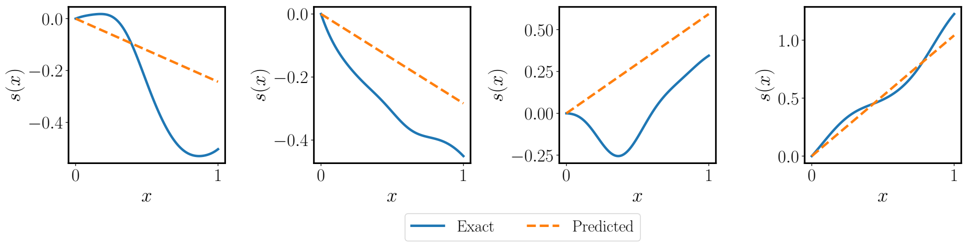

Figure S6: *Solving a 1D parametric ODE:* Exact solutions versus the predicted solutions of a trained DeepONet for four different input samples. We observe that the conventional DeepONet (*35*) learns a degenerate operator.

Model \ Relative L^2 error	Relative L^2 error of s	Relative L^2 error of u
DeepONet	$8.80e-01 \pm 4.72e-01$	$9.15e-01 \pm 1.86e-01$
Physics-informed DeepONet	$3.25e-03 \pm 3.19e-03$	$6.97e-03 \pm 3.95e-03$

Table S9: Solving a 1D parametric ODE: Mean and standard deviation of the relative L^2 prediction errors of a trained DeepONet and physics-informed DeepONet model over 1,000 examples in the test data-set.

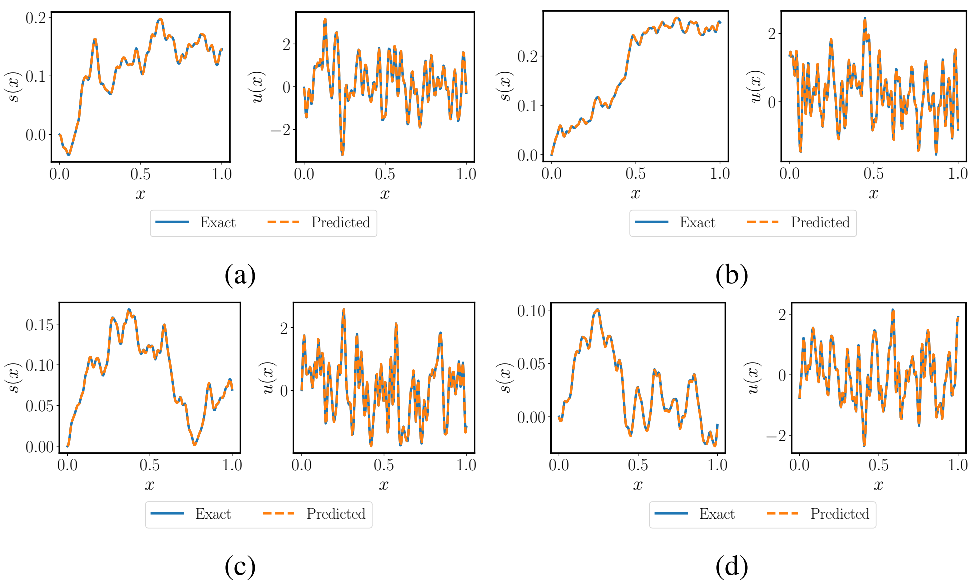

Figure S7: *Solving a 1D parametric ODE with irregular input functions:* Predicted solutions $s(x)$ and corresponding ODE residuals $u(x)$ for a trained physics-informed DeepONet with a with Fourier feature architecture, across four different examples in the test data-set.

Architecture \ Relative L^2 error	Relative L^2 error of s	Relative L^2 error of u
Fully-connected network	$3.48e-1 \pm 2.34e-1$	$6.81e-1 \pm 6.31e-2$
Fourier feature network	$8.45e-3 \pm 6.65e-3$	$8.25e-3 \pm 1.54e-3$

Table S10: *Solving a 1D parametric ODE with irregular input functions:* Mean and standard deviation of the relative L^2 prediction errors of physics-informed DeepONet represented by different network architectures over 1,000 examples in the test data-set.

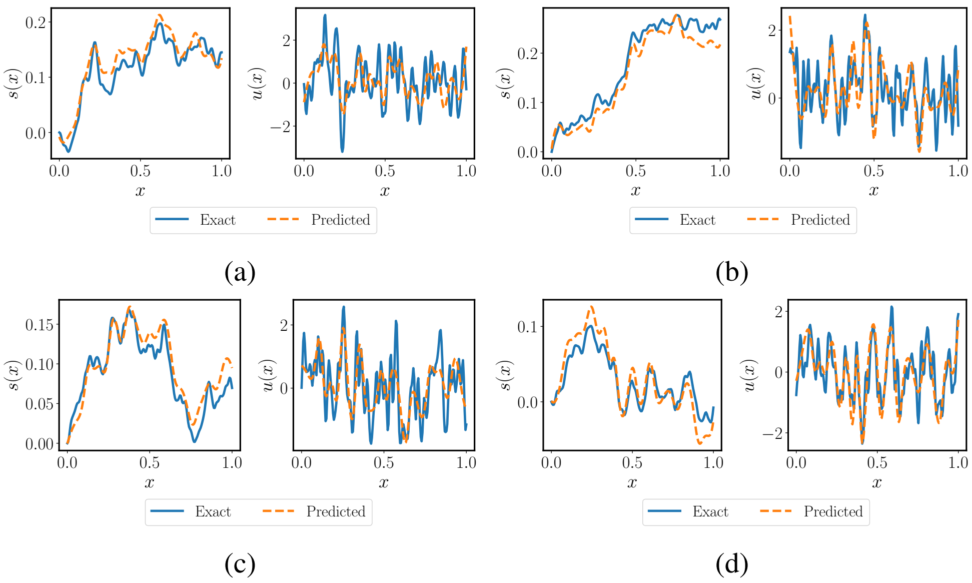

Figure S8: *Solving a 1D parametric ODE with irregular input functions:* Predicted solutions $s(x)$ and corresponding ODE residuals $u(x)$ for a trained physics-informed DeepONet with a conventional fully-connected architecture, across four different examples in the test data-set.

Diffusion-reaction system

Recall that the diffusion-reaction system is given by

$$\frac{\partial s}{\partial t} = D\frac{\partial^2 s}{\partial x^2} + ks^2 + u(x), \quad (x,t) \in (0,1] \times (0,1], \tag{35}$$

We approximate the operator by a physics-informed DeepONet architecture G_θ, where the branch and trunk networks are two separate 5-layer fully-connected neural networks with 50 neurons per hidden layer. For a given input function $\mathbf{u}^{(i)}$, we define the corresponding PDE residual as

$$R_\theta^{(i)}(x,t) = \frac{dG_\theta(\mathbf{u}^{(i)})(x,t)}{dt} - D\frac{d^2G_\theta(\mathbf{u}^{(i)})(x,t)}{dx^2} - k[G_\theta(\mathbf{u}^{(i)})(x,t)]^2, \tag{36}$$

where $\{\mathbf{u}^{(i)}\}_{i=1}^N = \{[u^{(i)}(x_1), u^{(i)}(x_2), \ldots, u^{(i)}(x_m)]\}_{i=1}^N$ represents the input functions, and $\{x_i\}_{i=1}^m$ is a collection of equi-spaced sensor locations in $[0,1]$. The parameters of the physics-

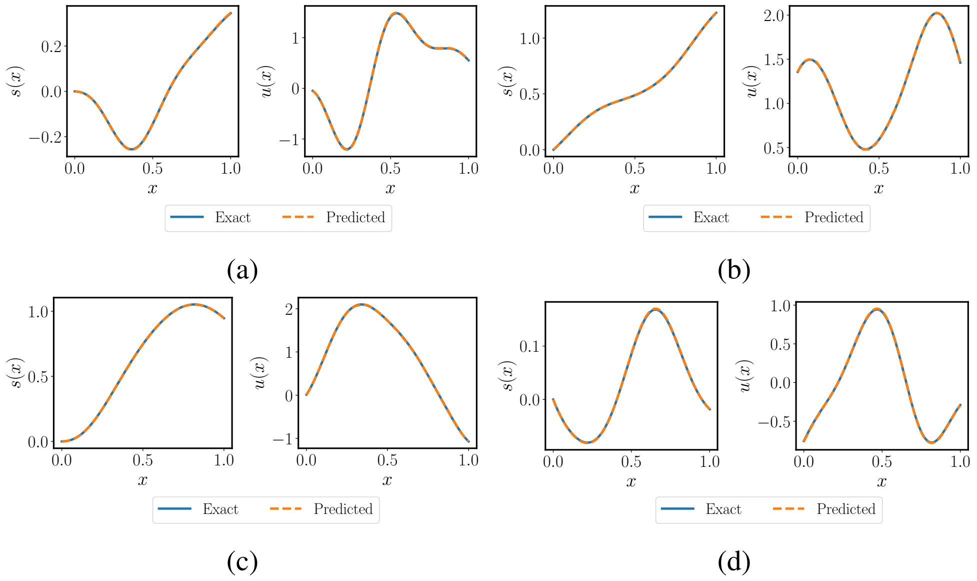

Figure S9: *Solving a 1D parametric ODE with irregular input functions:* Predicted solutions $s(x)$ and corresponding ODE residuals $u(x)$ for a trained physics-informed DeepONet with a with Fourier feature architecture, across four different out-of-distribution examples sampled from a GRF with a length scale $l = 0.2$ (recall that the training data for this case is generated using $l = 0.01$).

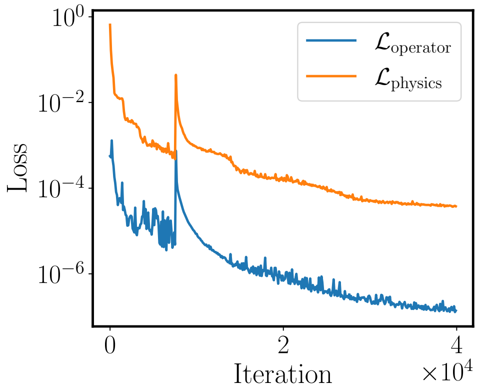

Figure S10: *Solving a 1D parametric ODE:* Training loss convergence of a physics-informed DeepONet for 40,000 iterations of gradient descent using the Adam optimizer without any paired input-output data, except the initial condition.

informed DeepONet can be trained by minimizing the loss function

$$\mathcal{L}(\theta) = \mathcal{L}_{\text{operator}}(\theta) + \mathcal{L}_{\text{physics}}(\theta) \tag{37}$$

$$= \frac{1}{NP} \sum_{i=1}^{N} \sum_{j=1}^{P} \left| G_\theta(\mathbf{u}^{(i)})(x_{u,j}^{(i)}, t_{u,j}^{(i)}) \right|^2 + \frac{1}{NQ} \sum_{i=1}^{N} \sum_{j=1}^{Q} \left| R_\theta^{(i)}(x_{r,j}^{(i)}, t_{r,j}^{(i)}) - u^{(i)}(x_{r,j}^{(i)}) \right|^2. \tag{38}$$

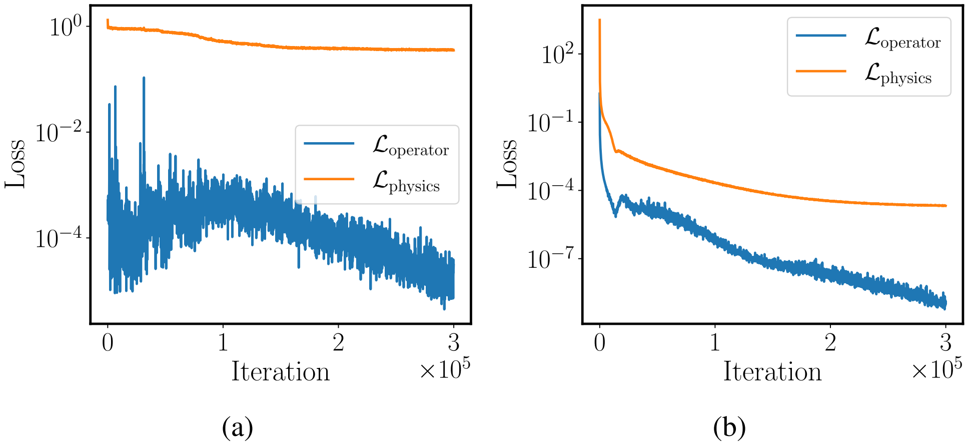

Figure S11: *Solving a 1D parametric ODE with irregular input functions:* (a)(b) Training loss convergence of a physics-informed DeepONets using a conventional fully-connected neural network, and a Fourier feature network, respectively, for 300,000 iterations of gradient descent using the Adam optimizer.

Here, for each $\mathbf{u}^{(i)}$, $\{(x_{u,j}^{(i)}, t_{u,j}^{(i)}\}_{j=1}^{P}$ are uniformly sampled points from the boundary of $[0,1] \times [0,1]$ (excluding $t = 1$), while $\{(x_{r,j}^{(i)}, t_{r,j}^{(i)})\}_{j=1}^{Q}$ is a set of collocation points satisfying $x_{r,j}^{(i)} = x_j$, and $\{t_{r,j}^{(i)})\}_{j=1}^{Q}$ are uniformly sampled in $[0,1]$. Consequently, $\mathcal{L}_{\text{operator}}(\theta)$ enforces the zero initial and boundary conditions, and $\mathcal{L}_{\text{physics}}(\theta)$ penalizes the parametric PDE residual at the Q collocation points. In this example, we set $P = Q = 100$ and randomly sample $N = 10,000$ input functions $u(x)$ from a GRF with length scale $l = 0.2$. To generate the test data-set, we sample $N = 1,000$ input functions $u(x)$ from the same GRF and solve the diffusion-reaction system using a second-order implicit finite difference method on a 100×100 equi-spaced grid (*71*). Hence, the test data-set will contain $1,000$ realizations evaluated on a 100×100 uniform grid. We train the physics-informed DeepONet by minimizing the loss function 37 for $120,000$ iterations of gradient descent using the Adam optimizer with default settings. Some visualizations for different input samples can be found in Appendix Figure S12.

To investigate the performance of a conventional DeepONet model (*35*) in the case where some training data are available. Specifically, we still use the same $10,000$ input functions

sampled before and for each u, and we randomly select $P = 100$ solution measurements out of the associated reference numerical solutions on the 100×100 grid. Then, we train the conventional DeepONet model under exactly the same hyper-parameter settings.

Furthermore, we study the effect of batch size in training physics-informed DeepONets. The resulting test error of training physics-informed DeepONets for different batch size are summarized in Figure S13. One may conclude that large batch size can effectively enhance the model predictive accuracy.

Finally, we perform a series of systematic studies to quantify the convergence of physics-informed DeepONets with respect to the number of input sensor locations, as well as the depth and the width of the branch and trunk networks. As shown in Figure S14, the test error generally decreases as the number of input sensor locations increases. A similar trend is observed also as the size of the network (i.e. the depth and width of the branch and trunk network) increases. In particular, increasing the depth of the network tends to yield better predictive accuracy than increasing the width. In addition, the number of sensors should be enough to capture all necessary frequency information of the input functions. The smaller the length scale of the GRF, the larger number of sensors are required.

Burgers' equation

The governing law takes the form

$$\frac{ds}{dt} + s\frac{ds}{dx} - \nu\frac{d^2 s}{dx^2} = 0, \quad (x,t) \in (0,1) \times (0,1], \tag{39}$$

$$s(x,0) = u(x), \quad x \in (0,1), \tag{40}$$

subject to periodic boundary conditions

$$s(0,t) = s(1,t), \tag{41}$$

$$\frac{ds}{dx}(0,t) = \frac{ds}{dx}(1,t), \tag{42}$$

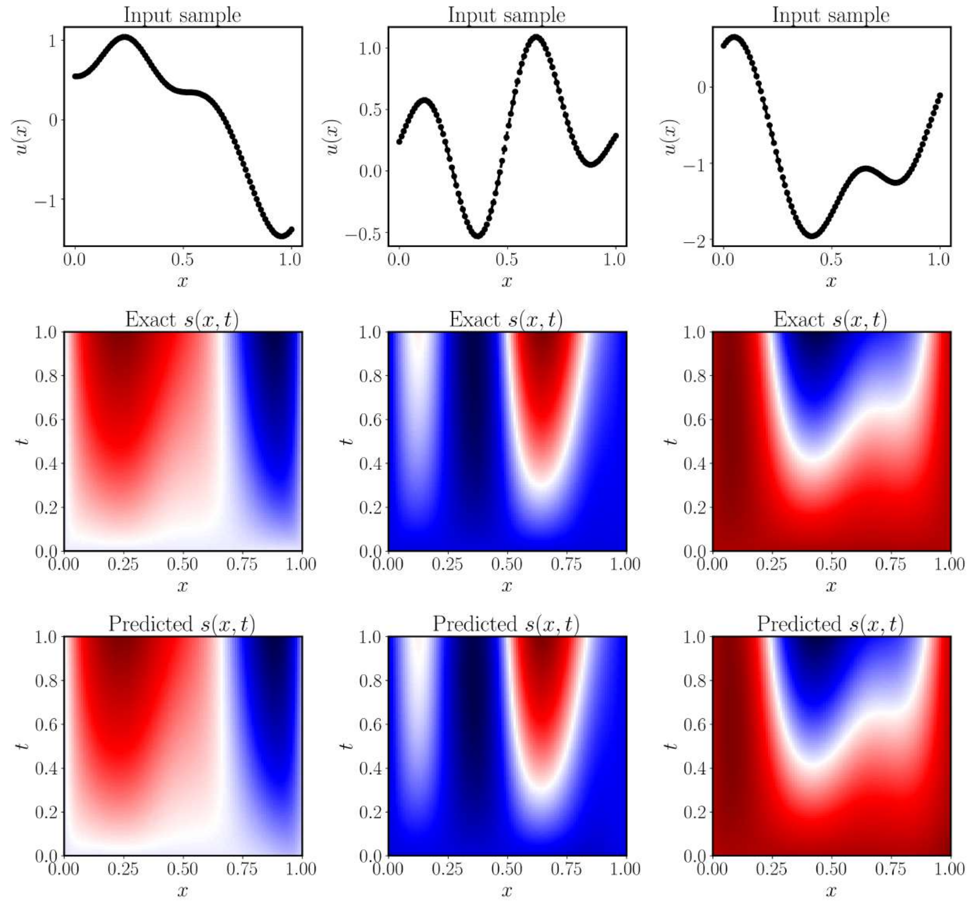

Figure S12: *Solving a parametric diffusion-reaction system:* Predicted solution of a trained physics-informed DeepONet for three different examples in the test data-set.

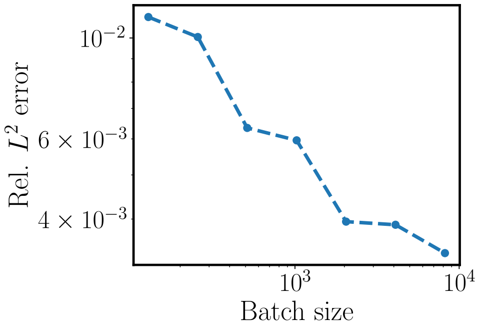

Figure S13: *Solving a parametric diffusion-reaction system:* Relative L^2 prediction error of physics-informed DeepONets trained using a different batch-size, averaged over 1,000 examples in the test data-set.

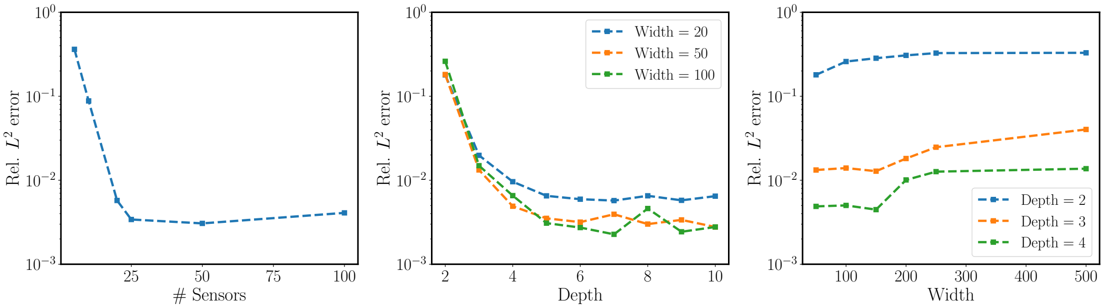

(a) (b) (c)

Figure S14: *Solving a parametric diffusion-reaction system:* (a)(b)(c) Relative L^2 prediction error of physics-informed DeepONets trained using: (a) different number of input sensors; (b, c) depth and width of the branch and trunk networks, respectively, averaged over 1,000 examples in the test data-set.

Suppose that the solution operator is approximated by a physics-informed DeepONet G_θ. For a specific input function $\mathbf{u}^{(i)}$, the PDE residual is defined by

$$R_\theta^{(i)}(x,t) = \frac{dG_\theta(\mathbf{u}^{(i)})(x,t)}{dt} + G_\theta(\mathbf{u}^{(i)})(x,t)\frac{dG_\theta(\mathbf{u}^{(i)})(x,t)}{dx} - \nu\frac{d^2G_\theta(\mathbf{u}^{(i)})(x,t)}{dx^2}, \tag{43}$$

where $\mathbf{u}^{(i)}$ denotes the input function evaluated a collection of fixed sensors $\{x_i\}_{i=1}^m$ that are uniformly spaced in $[0,1]$. Then, the physics-informed loss function is given by

$$\mathcal{L}(\theta) = \mathcal{L}_{\text{IC}}(\theta) + \mathcal{L}_{\text{BC}}(\theta) + \mathcal{L}_{\text{physics}}(\theta), \tag{44}$$

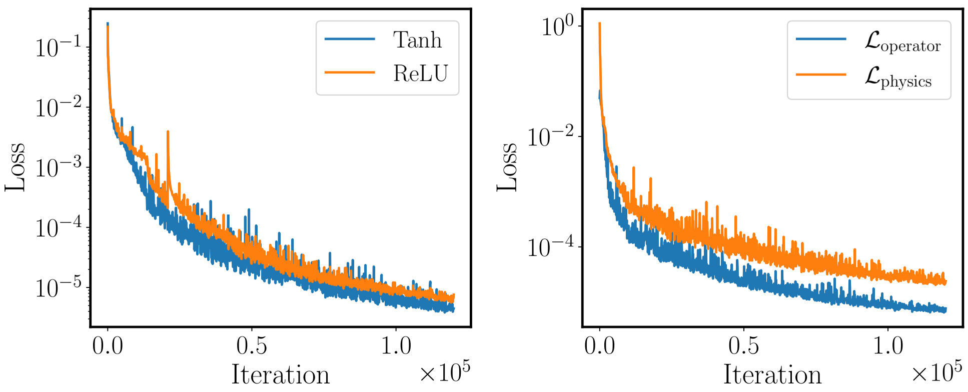

(a) (b)

Figure S15: *Solving a parametric diffusion-reaction system:* (a) Training loss convergence of a DeepONet equipped with different activations for 120,000 iterations of gradient descent using the Adam optimizer (with paired input-output training data). (b) Training loss convergence of a physics-informed DeepONet equipped with Tanh activations for 120,000 iterations of gradient descent using the Adam optimizer (without paired input-output training data).

where

$$\mathcal{L}_{\text{IC}}(\theta) = \frac{1}{NP}\sum_{i=1}^{N}\sum_{j=1}^{P}\left|G_\theta(\mathbf{u}^{(i)})(x_{ic,j}^{(i)}, 0) - u^{(i)}(x_{ic,j}^{(i)})\right|^2 \tag{45}$$

$$\mathcal{L}_{\text{BC}}(\theta) = \frac{1}{NP}\sum_{i=1}^{N}\sum_{j=1}^{P}\left|G_\theta(\mathbf{u}^{(i)})(0, t_{bc,j}^{(i)}) - G_\theta(\mathbf{u}^{(i)})(1, t_{bc,j}^{(i)})\right|^2 \tag{46}$$

$$+ \frac{1}{NP}\sum_{i=1}^{N}\sum_{j=1}^{P}\left|\frac{dG_\theta(\mathbf{u}^{(i)})(x,t)}{dx}\bigg|_{(0,t_{bc,j}^{(i)})} - \frac{dG_\theta(\mathbf{u}^{(i)})(x,t)}{dx}\bigg|_{(1,t_{bc,j}^{(i)})}\right|^2 \tag{47}$$

$$\mathcal{L}_{\text{physics}}(\theta) = \frac{1}{NQ}\sum_{i=1}^{N}\sum_{j=1}^{Q}\left|R_\theta^{(i)}(x_{r,j}^{(i)}, t_{r,j}^{(i)})\right|^2. \tag{48}$$

Here, for every input sample $\mathbf{u}^{(i)}$, $x_{ic,j}^{(i)} = x_j$ and $\{(0, t_{ic,j}^{(i)})\}_{j=1}^{P}, \{(1, t_{ic,j}^{(i)})\}_{j=1}^{P}$ and $\{(x_{r,j}^{(i)}, t_{r,j}^{(i)})\}_{j=1}^{Q}$ are randomly sampled in the computational domain for enforcing the initial and boundary conditions and the PDE residual, respectively. In this example, we take $P = m = 100, Q = 2,500$.

To obtain a set of training and test data, we randomly sample 2,000 input functions from a GRF $\sim \mathcal{N}(0, 25^2(-\Delta + 5^2 I)^{-4})$, and select a subset of $N = 1,000$ samples as training data. For each sample u, we solve the Burgers equation 39 using conventional spectral methods.

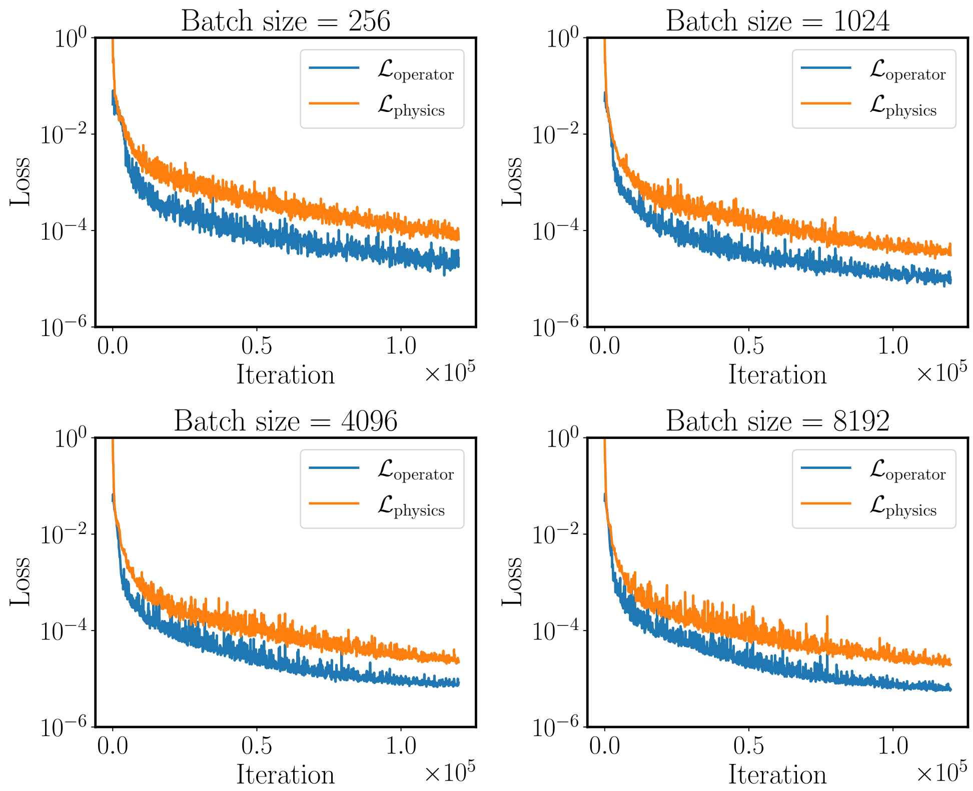

Figure S16: *Solving a parametric diffusion-reaction system:* Training loss convergence of physics-informed DeepONets trained using a different batch-size.

Specifically, assuming periodic boundary conditions, we start from a given initial condition $s(x, 0) = u(x), x \in [0, 1]$ and integrate the equation 39 up to the final time $t = 1$. Synthetic test data for this example are generated using the Chebfun package (*58*) with a spectral Fourier discretization and a fourth-order stiff time-stepping scheme (ETDRK4) (*74*) with time-step size 10^{-4}. Temporal snapshots of the solution are saved every $\Delta t = 0.01$ to give us 101 snapshots in total. Consequently, the test data-set contains 1,000 realizations evaluated at a 100×101 spatio-temporal grid.

We employ two separate 7-layer fully-connected neural networks to represent the branch net and the trunk net, respectively. Each network is equipped with Tanh activation functions and has 100 units per hidden layer. The physics-informed DeepONet is trained by minimizing

the loss function 44 via gradient descent using the Adam optimizer for $200,000$ iterations. Figure S17c shows the predicted solution of a trained physics-informed DeepONet for the worst sample in the test data-set, with a resulting relative L^2 error of 17%. Moreover, a discrepancy between the exact and the predicted initial condition $u(x)$ can be observed in Figure S17a. This indicates that the physics-informed DeepONet cannot accurately reconstruct the initial condition, which results in a large prediction error of the full solution. To enforce the initial condition and improve the performance of physics-informed DeepONet, we consider assigning weights to $\mathcal{L}_{IC}(\theta)$ and use a more powerful network architecture as the backbone of the branch net and the trunk net. Specifically, we modify the loss function 44 as

$$\mathcal{L}(\theta) = \lambda\mathcal{L}_{\text{IC}}(\theta) + \mathcal{L}_{\text{BC}}(\theta) + \mathcal{L}_{\text{physics}}(\theta), \tag{49}$$

where λ is a hyper-parameter that aims to balance the interplay of different terms in the loss function. Moreover, we employ a simple modified fully-connected neural network proposed by Wang *et. al* (*62*), which has been empirically proven to outperform conventional multi-layer percerptron (MLP) networks. Specifically, the forward pass of the proposed modified MLP architecture is given by

$$U = \phi(XW^1 + b^1), \;\; V = \phi(XW^2 + b^2) \tag{50}$$

$$H^{(1)} = \phi(XW^{z,1} + b^{z,1}) \tag{51}$$

$$Z^{(k)} = \phi(H^{(k)}W^{z,k} + b^{z,k}), \;\; k = 1, \ldots, L \tag{52}$$

$$H^{(k+1)} = (1 - Z^{(k)}) \odot U + Z^{(k)} \odot V, \;\; k = 1, \ldots, L \tag{53}$$

$$f_\theta(x) = H^{(L+1)}W + b, \tag{54}$$

where X denotes the network inputs, and $\odot$ denotes element-wise multiplication. The parameters of this model are essentially the same as in a standard fully-connected architecture, with

the addition of the weights and biases used by the two transformer networks, i.e.,

$$\theta = \{W^1, b^1, W^2, b^2, (W^{z,l}, b^{z,l})_{l=1}^L, W, b\} \tag{55}$$

We train the physics-informed DeepONet using standard and modified MLP networks by minimizing the modified loss function 49 for different $\lambda \in \{1, 5, 10, 20, 50, 100\}$ under exactly the same hyper-parameter setting. The resulting average relative L^2 prediction errors are summarized in Figure S18a. Compared against conventional MLPs, the modified MLP architecture is capable of consistently yielding much better prediction accuracy, which can be further improved by assigning appropriate weights in the loss function. Among all these hyper-parameters, the smallest test error $\sim 1.38\%$ is obtained for the modified fully-connected neural network with $\lambda = 20$. More visualizations for different input samples are shown in Figure S19.

We also investigate the effect of the viscosity parameter in the Burgers equation on the performance of physics-informed DeepONets. In particular, we vary the viscosity ν from 10^{-2} to 10^{-4} and train physics-informed DeepONets under exactly the same hyper-parameter settings (network architecture, learning rate, batch size, weights, etc). As shown in Figure S20 the predicted solutions are in qualitative agreement with the corresponding ground truth. However, one can observe a large discrepancy in regions where the solution exhibits steep gradients. This is further validated by the averaged relative L^2 error reported in Table S11. This result is in agreement with our previous experience, as well as the PINNs literature, and confirms the difficulties that deep learning approaches face in presence of stiff dynamics. While our results appear qualitatively promising, there is certainly a need for further methodological advances for enhancing the accuracy and robustness of physics-informed DeepONets in this setting. We believe that these issues will be tackled in the future by designing of more specialized architectures, as well as more effective optimization algorithms for training constrained neural network models, such as PINNs and physics-informed DeepONets.

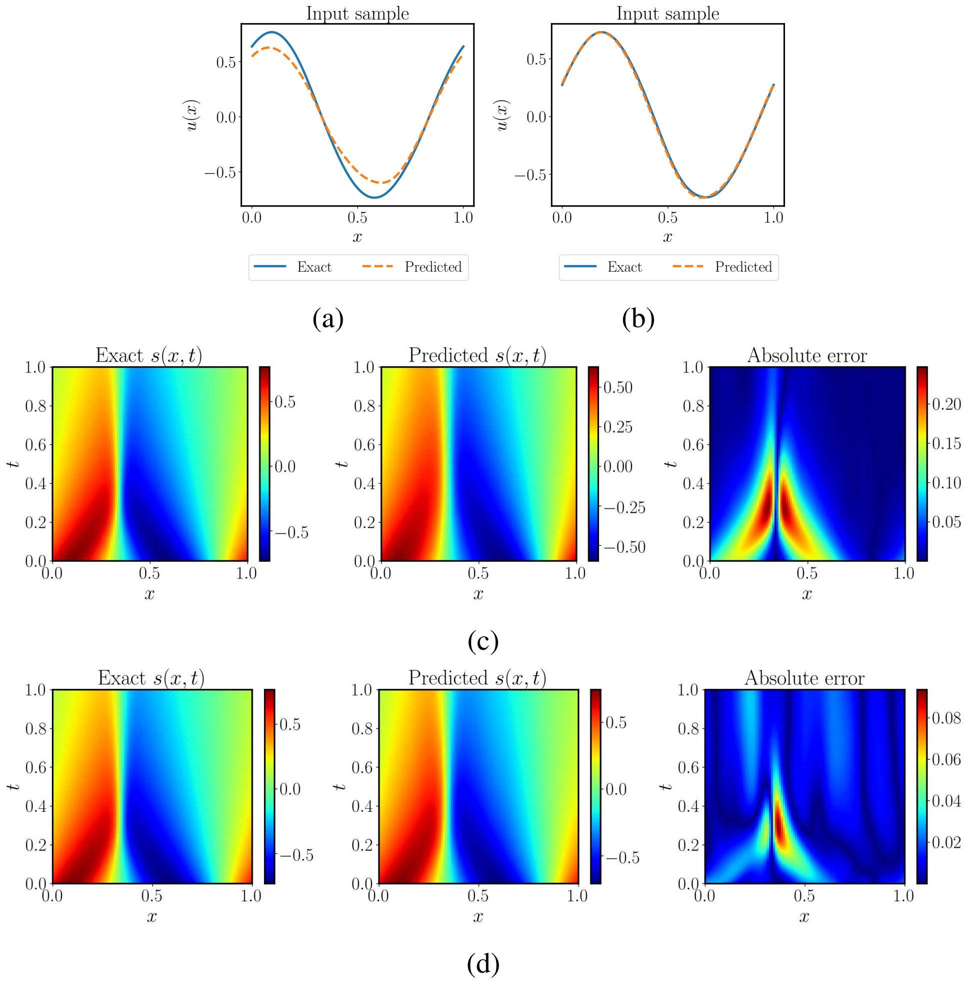

Figure S17: *Solving a parametric Burgers' equation:* (a)(c) Exact solution and the initial condition versus the predictions of a trained physics-informed DeepONet with a conventional MLP architecture. The resulting relative L^2 error of the predicted solution is 17.1%. (b)(d) Exact solution and the initial condition versus the predictions of a trained physics-informed DeepONet with a modified MLP architecture (*62*) and $\lambda = 20$. The resulting relative L^2 error of the predicted solution is reduced to 3%.

Advection equation

To further investigate the performance of physics-informed DeepOnets in advection-dominated problems, we have considered an additional benchmark involving a simple hyperbolic PDE

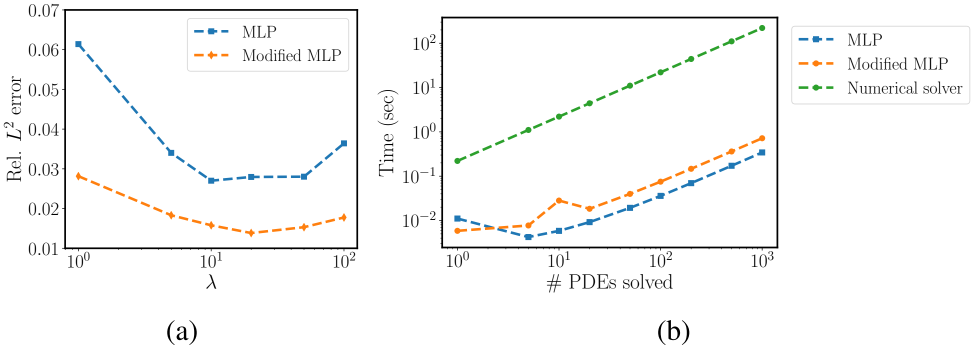

Figure S18: *Solving a parametric Burgers' equation:* (a) The average relative L^2 error of training physics-informed DeepONets with standard or modified MLPs for different $\lambda \in \{1, 5, 10, 20, 50, 100\}$ over 1,000 examples in the test data-set. The smallest errors for standard and modified MLPs are 2.8% and 1.3%, respectively. (b) Computational cost (sec) for performing inference with a trained physics-informed DeepONet model (conventional or modified MLP architecture), as well as corresponding timing for solving a PDE with a conventional spectral solver (*58*). Strikingly, a trained physics informed DeepOnet model can predict the solution of $\mathcal{O}(10^3)$ time-dependent PDEs in a fraction of a second – up to three orders of magnitude faster compared to a conventional PDE solver. Reported timings are obtained on a single NVIDIA V100 GPU.

Viscosity	$\nu = 0.01$	$\nu = 0.001$	$\nu = 0.0001$
Rel. L^2 error	$1.38\% \pm 1.64\%$	$21.62\% \pm 14.45\%$	$24.76\% \pm 10.69\%$

Table S11: *Solving a parametric Burger's equation:* Relative L^2 prediction error of a trained physics-informed DeepONet averaged over all examples in the test data-set, for different viscosity values.

system. Specifically, we have considered the following parametric advection problem

$$\frac{\partial s}{\partial t} + u(x)\frac{\partial s}{\partial x} = 0, \quad (x,t) \in (0,1) \times (0,1), \tag{56}$$

with the initial and boundary condition

$$s(x,0) = f(x) \tag{57}$$

$$s(0,t) = g(t) \tag{58}$$

where $f(x) = \sin(\pi x)$ and $g(t) = \sin(\frac{\pi}{2}t)$. To make the input function $u(x)$ strictly positive, we let $u(x) = v(x) - \min_x v(x) + 1$ where $v(x)$ is sampled from a GRF with a length scale

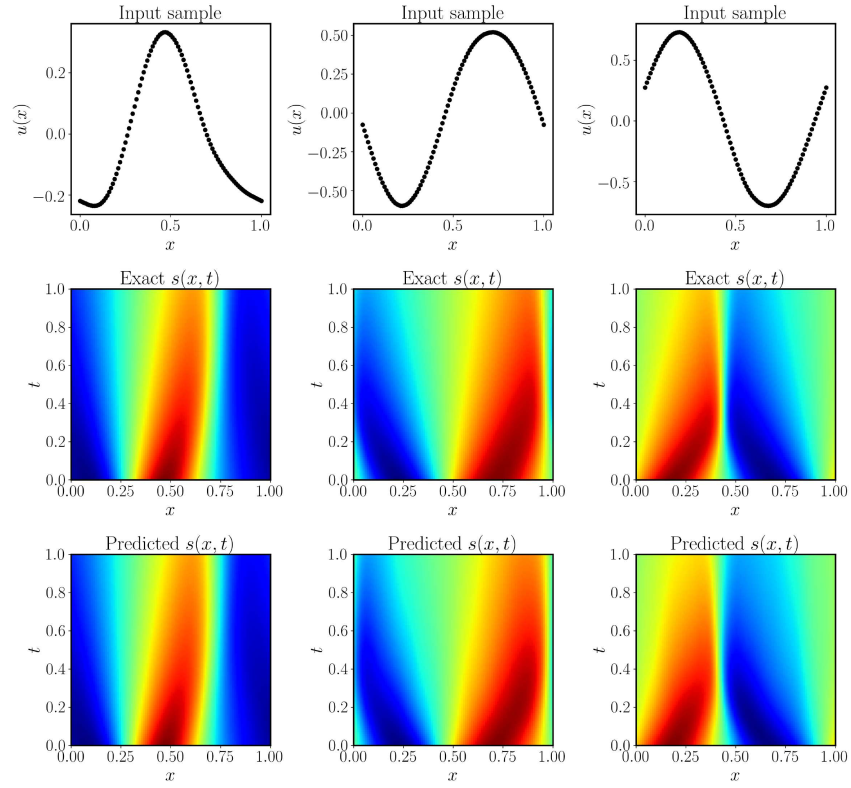

Figure S19: *Solving a Burgers' equation:* Predicted solutions of a trained physics-informed DeepONet with a modified MLP architecture for three different examples in the test data-set.

$l = 0.2$. The goal is to learn the solution operator G mapping variable coefficients $u(x)$ to the associated PDE solution $s(x,t)$. A physics-informed loss can be formulated as follows

$$\mathcal{L}(\theta) = \mathcal{L}_{\text{BC}}(\theta) + \mathcal{L}_{\text{IC}}(\theta) + \mathcal{L}_{\text{physics}}(\theta), \tag{59}$$

where

$$\mathcal{L}_{\text{IC}}(\theta) = \frac{1}{NP}\sum_{i=1}^{N}\sum_{j=1}^{P}\left|G_\theta(\mathbf{u}^{(i)})(x_{ic,j}^{(i)}, 0) - f(x_{ic,j}^{(i)})\right|^2, \tag{60}$$

$$\mathcal{L}_{\text{BC}}(\theta) = \frac{1}{NP}\sum_{i=1}^{N}\sum_{j=1}^{P}\left|G_\theta(\mathbf{u}^{(i)})(0, t_{bc,j}^{(i)}) - g(t_{bc,j}^{(i)})\right|^2, \tag{61}$$

$$\mathcal{L}_{\text{physics}}(\theta) = \frac{1}{NQ}\sum_{i=1}^{N}\sum_{j=1}^{Q}\left|\frac{\partial G_\theta(\mathbf{u}^{(i)})(x_{r,j}^{(i)}, t_{r,j}^{(i)})}{\partial t} - u^{(i)}(x_{r,j}^{(i)})\frac{\partial G_\theta(\mathbf{u}^{(i)})(x_{r,j}^{(i)}, t_{r,j}^{(i)})}{\partial x}\right|^2. \tag{62}$$

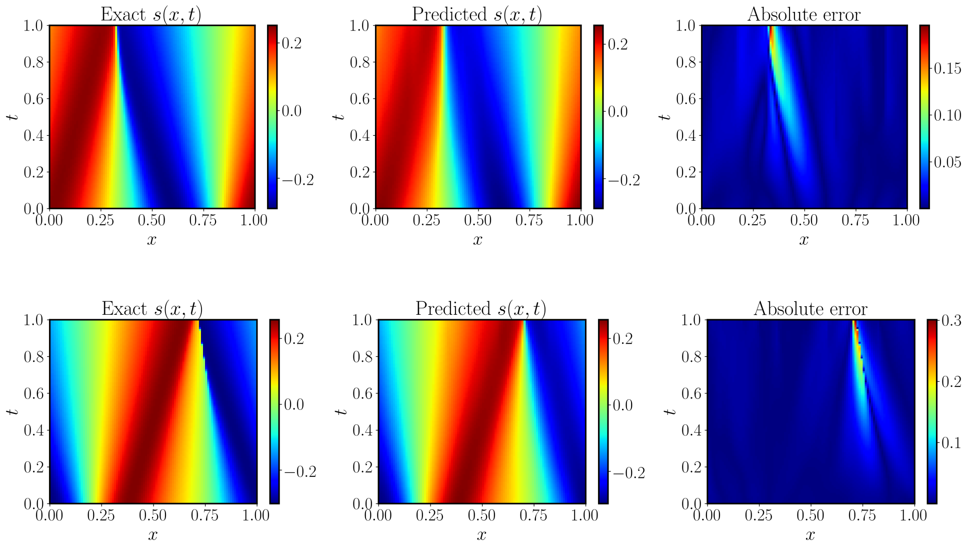

Figure S20: *Solving a parametric Burger's equation:* Predictions of a trained physics-informed DeepONet for two representative initial conditions in the test data-set, corresponding to different viscosity values of $\nu = 10^{-3}$ (*top*), and 10^{-4} (*bottom*), respectively.

Here, we take $N = 2,000$, $P = 200$ and $Q = 2,500$. The input sample $\mathbf{u}^{(i)}$ is evaluated at equi-spaced points $\{x_i\}_{i=1}^m$ in $[0,1]$. To generate a set of test data, we sample $N = 100$ input functions from the same GRF and solve the advection equation using the Lax–Wendroff scheme (*71*) on a 100×100 uniform grid.

The DeepOnet architecture consists of two modified fully-connected neural networks as the branch and trunk network, respectively. Each network has 7 layers and 100 neurons per hidden layer. We train the physics-informed DeepONet by minimizing the above loss function for $300,000$ iterations. The resulting relative L^2 errors of the trained model over the test data-set is 2.24%. Representative visualizations of the predicted solution for different input samples are shown in Figure 5. We see that the predictions achieve a good agreement with the numerical estimations overall, while some inaccuracies can be founded near the fast transition in the ground truth. We believe that this is because the DeepONet cannot accurately approximate the sharp gradients during training, which is also consistent with our observations for the Burgers'

equation with small viscosity.

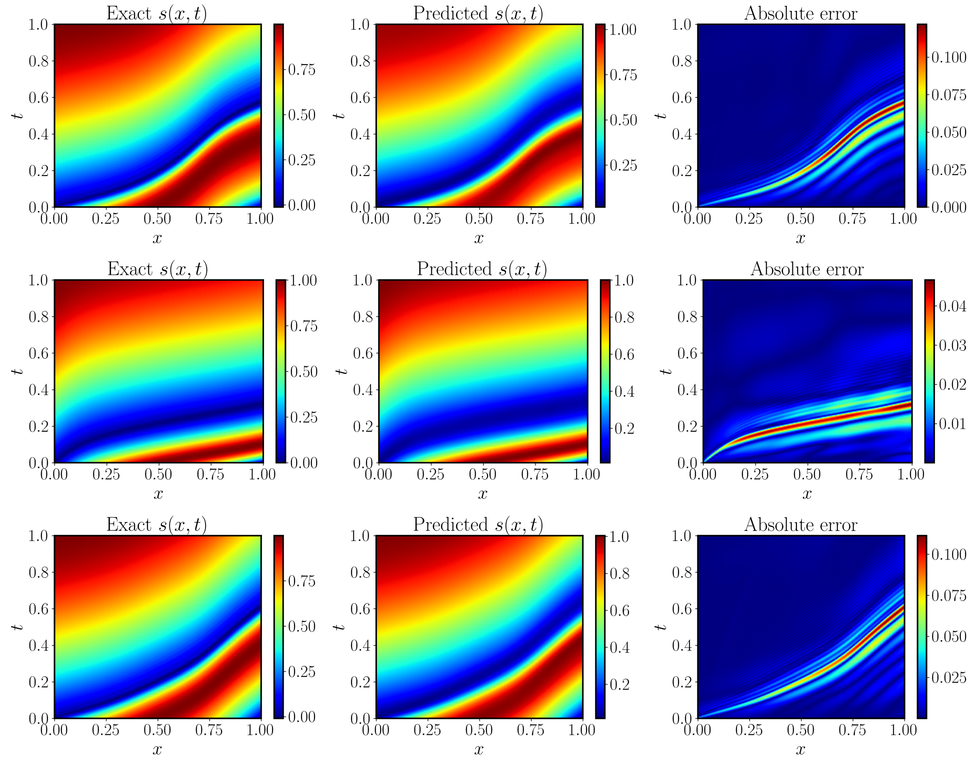

Figure S21: *Solving parametric a advection equation:* Predicted solutions of a trained physics-informed DeepONet for three examples in the test data-set.

Eikonal equation

Recall that the Eikonal equation is given by

$$\|\nabla s(\mathbf{x})\|_2 = 1, \qquad s(\mathbf{x}) = 0, \quad \mathbf{x} \in \partial\Omega, \tag{63}$$

We use a DeepONet G_θ to represent the unknown operator. This allows us to define the PDE residual

$$R_\theta^{(i)}(x,y) = \|\nabla G_\theta(\mathbf{\Gamma}^{(i)})(x,y)\|_2 = \left\| \sqrt{\left(\frac{dG_\theta(\mathbf{\Gamma}^{(i)})(x,y)}{dx}\right)^2 + \left(\frac{dG_\theta(\mathbf{\Gamma}^{(i)})(x,y)}{dy}\right)^2} \right\|_2 \tag{64}$$

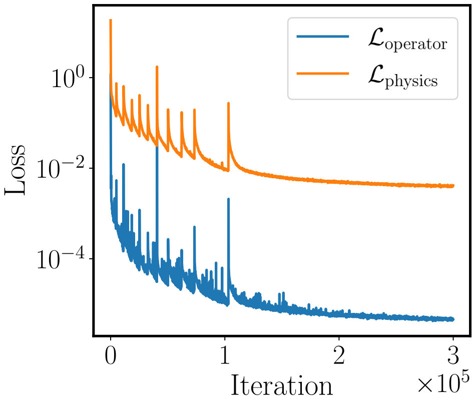

Figure S22: *Solving a parametric advection equation:* Training loss convergence of a physics-informed DeepONet over $300,000$ training iterations with gradient descent using the Adam optimizer.

Here, $\mathbf{\Gamma}^{(i)} = [(x_1^{(i)}, y_1^{(i)}), (x_2^{(i)}, y_2^{(i)}), \dots, (x_m^{(i)}, y_m^{(i)})]$ denotes a parametrized curve evaluated at a set of fixed sensor locations $\{(x_j^{(i)}, y_j^{(i)})\}_{j=1}^m$. Then, a physics-informed DeepONet can be trained by minimizing the following loss function

$$\mathcal{L}(\theta) = \mathcal{L}_{\text{BC}}(\theta) + \mathcal{L}_{\text{physics}}(\theta) \tag{65}$$

$$= \frac{1}{Nm} \sum_{i=1}^{N} \sum_{j=1}^{m} \left| G_\theta(\mathbf{\Gamma}^{(i)})(x_j^{(i)}, y_j^{(i)}) \right|^2 + \frac{1}{NQ} \sum_{i=1}^{N} \sum_{j=1}^{Q} \left| R_\theta^{(i)}(x_{r,j}^{(i)}, y_{r,j}^{(i)}) - 1 \right|^2, \tag{66}$$

where $\mathcal{L}_{\text{BC}}(\theta)$ and $\mathcal{L}_{\text{physics}}(\theta)$ are used to impose the zero boundary condition and the PDE residual, respectively. Moreover, for each input curve $\mathbf{\Gamma}^{(i)}$, $\{(x_{r,j}^{(i)}, y_{r,j}^{(i)})\}_{j=1}^Q$ are uniformly sampled in the given computational domain. Unlike the previous parametric PDE examples, it is worth noting that the "input functions" of this example are actually defining a variable computational domain.

Case I: Circles

We start with a simple case corresponding to circular boundaries $\partial\Omega$ centered at the origin, each having a different radius. In this case, the corresponding signed distance function that solves equation 63 can be analytically derived. For example, suppose that Γ is a circle with radius r,

then the signed distance function is given by

$$s(x, y) = \sqrt{x^2 + y^2} - r \tag{67}$$

To generate a set of training data, we randomly choose $N = 1,000$ circles with radii sampled from a uniform distribution. Then, for each input circle $\Gamma^{(i)}$ with radius $r^{(i)}$, we have $\{(x_j^{(i)}, y_j^{(i)})\}_{j=1}^Q = \{(r^{(i)} \cos\theta_j, r^{(i)} \sin\theta_j)\}_{j=1}^Q$, where $\{\theta_j\}_{j=1}^Q$ are evenly spaced in $[0, 2\pi]$. Here, we consider a computational domain $D = [-2, 2] \times [-2, 2]$ and we set $m = 100, Q = 1,000$.

The branch net and the trunk networks are two separate 6-layer fully-connected neural network with 50 neurons per hidden layer. We train the physics-informed DeepONet by minimizing the loss function 65 for $80,000$ iterations of gradient descent using the Adam optimizer. As shown in Figure S23, an excellent agreement can be achieved between the exact and the predicted signed distance functions for a representative example in the test data-set. The relative L^2 prediction error averaged over 1,000 examples in the test data-set is 0.4%. More model predictions for different input samples can be found in Figure S24.

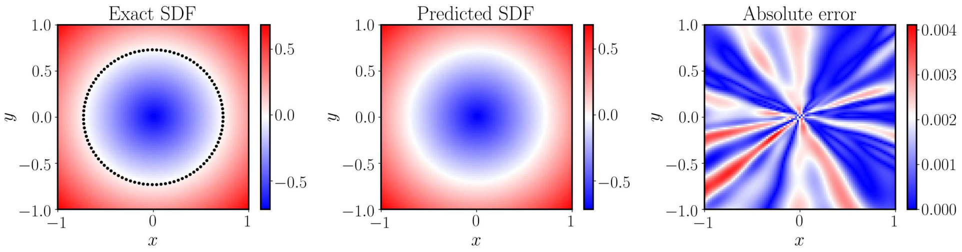

Figure S23: *Solving a parametric Eikonal equation (circles):* Exact solutions versus the predicted solutions of a trained physics-informed DeepONet for a representative input sample. The black dots represent the location of sensors on the circular boundary.

Case II: Airfoils

Below, we describe some details of the data-set and the training procedure. To obtain a set of training and test data, we use the UIUC Airfoil Data Site (*60*) which contains a total of 1,552

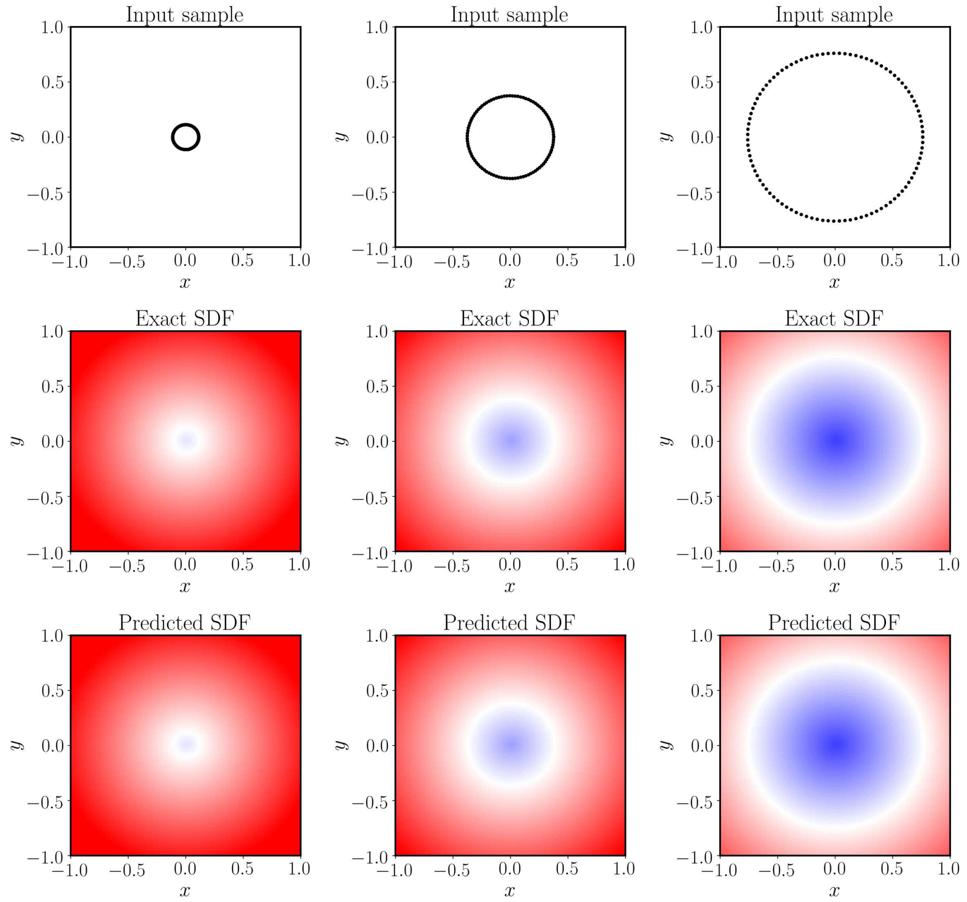

Figure S24: *Solving a parametric Eikonal equation (circles):* Predicted signed distance functions by a trained physics-informed DeepONet for three different examples in the test data-set.

airfoil geometries. We use the first 1,000 shapes as training data, and the rest are included in the test data-set. Without loss of generality, we normalize the airfoil shapes to have zero mean and unit variance.

In this example, the computation domain is the unit square $[-3, 3] \times [-3, 3]$ and the branch net and the trunk net are two separate 6-layer fully-connected neural networks with 100 neurons per hidden layer. Both two networks are equipped with ELU activation functions. We train the physics-informed DeepONet for $120,000$ iterations of gradient descent using the Adam optimizer.

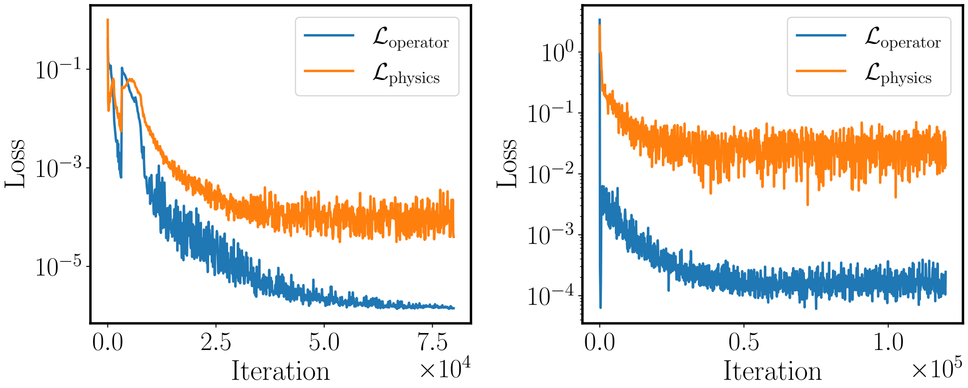

(a) (b)

Figure S25: *Solving a parametric Eikonal equation:* (a) Training loss convergence of a physics-informed DeepONet equipped with Tanh activations, for 80,000 iterations of gradient descent using the Adam optimizer. (b) Training loss convergence of a physics-informed DeepONet equipped with ELU activations, for 120,000 iterations of gradient descent using the Adam optimizer.

REFERENCES AND NOTES

1. R. Courant, D. Hilbert, *Methods of Mathematical Physics: Partial Differential Equations* (John Wiley & Sons, 2008).

2. T. J. Hughes, *The Finite Element Method: Linear Static and Dynamic Finite Element Analysis* (Courier Corporation, 2012).

3. D. J. Lucia, P. S. Beran, W. A. Silva, Reduced-order modeling: New approaches for computational physics. *Prog. Aerosp. Sci.* **40**, 51–117 (2004).

4. J. N. Kutz, S. L. Brunton, B. W. Brunton, J. L. Proctor, *Dynamic Mode Decomposition: Data-Driven Modeling of Complex Systems* (SIAM, 2016).

5. P. Benner, M. Ohlberger, A. Patera, G. Rozza, K. Urban, *Model Reduction of Parametrized Systems* (Springer, 2017).

6. W. H. Schilders, H. A. Van der Vorst, J. Rommes, in *Model Order Reduction: Theory, Research Aspects and Applications* (Springer, 2008), vol. 13.

7. A. Quarteroni, G. Rozza, in *Reduced Order Methods for Modeling and Computational Reduction* (Springer, 2014), vol. 9.

8. I. Mezić, Spectral properties of dynamical systems, model reduction and decompositions. *Nonlinear Dyn.* **41**, 309–325 (2005).

9. B. Peherstorfer, K. Willcox, Data-driven operator inference for nonintrusive projection-based model reduction. *Comput. Methods Appl. Mech. Eng.* **306**, 196–215 (2016).

10. A. J. Majda, D. Qi, Strategies for reduced-order models for predicting the statistical responses and uncertainty quantification in complex turbulent dynamical systems. *SIAM Rev.* **60**, 491–549 (2018).

11. T. Lassila, A. Manzoni, A. Quarteroni, G. Rozza, Model order reduction in fluid dynamics: Challenges and perspectives, in *Reduced Order Methods for Modeling and Computational Reduction* (Springer, 2014), pp. 235–273.

12. D. C. Psichogios, L. H. Ungar, A hybrid neural network-first principles approach to process modeling. *AIChE J.* **38**, 1499–1511 (1992).

13. I. E. Lagaris, A. Likas, D. I. Fotiadis, Artificial neural networks for solving ordinary and partial differential equations. *IEEE Trans. Neural Netw.* **9**, 987–1000 (1998).

14. M. Raissi, P. Perdikaris, G. E. Karniadakis, Physics-informed neural networks: A deep learning framework for solving forward and inverse problems involving nonlinear partial differential equations. *J. Comput. Phys.* **378**, 686–707 (2019).

15. L. Sun, H. Gao, S. Pan, J.-X. Wang, Surrogate modeling for fluid flows based on physics-constrained deep learning without simulation data. *Comput. Methods Appl. Mech. Eng.* **361**, 112732 (2020).

16. Y. Zhu, N. Zabaras, P.-S. Koutsourelakis, P. Perdikaris, Physics-constrained deep learning for high-dimensional surrogate modeling and uncertainty quantification without labeled data. *J. Comput. Phys.* **394**, 56–81 (2019).

17. S. Karumuri, R. Tripathy, I. Bilionis, J. Panchal, Simulator-free solution of high-dimensional stochastic elliptic partial differential equations using deep neural networks. *J. Comput. Phys.* **404**, 109120 (2020).

18. J. Sirignano, K. Spiliopoulos, DGM: A deep learning algorithm for solving partial differential equations. *J. Comput. Phys.* **375**, 1339–1364 (2018).

19. M. Raissi, A. Yazdani, G. E. Karniadakis, Hidden fluid mechanics: Learning velocity and pressure fields from flow visualizations. *Science* **367**, 1026–1030 (2020).

20. A. Tartakovsky, C. O. Marrero, P. Perdikaris, G. Tartakovsky, D. Barajas-Solano, Physics-informed deep neural networks for learning parameters and constitutive relationships in subsurface flow problems. *Water Resour. Res.* **56**, e2019WR026731 (2020).

21. O. Hennigh, S. Narasimhan, M. A. Nabian, A. Subramaniam, K. Tangsali, M. Rietmann, J. del Aguila Ferrandis, W. Byeon, Z. Fang, S. Choudhry, NVIDIA SimNet: An ai-accelerated multi-physics simulation framework. arXiv:2012.07938 (2020).

22. S. Cai, Z. Wang, S. Wang, P. Perdikaris, G. Karniadakis, Physics-informed neural networks (pinns) for heat transfer problems. *J. Heat Transfer* **143**, 060801 (2021).

23. G. Kissas, Y. Yang, E. Hwuang, W. R. Witschey, J. A. Detre, P. Perdikaris, Machine learning in cardiovascular flows modeling: Predicting arterial blood pressure from non-invasive 4D flow MRI data using physics-informed neural networks. *Comput. Methods Appl. Mech. Eng.* **358**, 112623 (2020).

24. F. Sahli Costabal, Y. Yang, P. Perdikaris, D. E. Hurtado, E. Kuhl, Physics-informed neural networks for cardiac activation mapping. *Front. Phys.* **8**, 42 (2020).

25. L. Lu, M. Dao, P. Kumar, U. Ramamurty, G. E. Karniadakis, S. Suresh, Extraction of mechanical properties of materials through deep learning from instrumented indentation. *Proc. Natl. Acad. Sci. U.S.A.* **117**, 7052–7062 (2020).

26. Y. Chen, L. Lu, G. E. Karniadakis, L. Dal Negro, Physics-informed neural networks for inverse problems in nano-optics and metamaterials. *Opt. Express* **28**, 11618–11633 (2020).

27. S. Goswami, C. Anitescu, S. Chakraborty, T. Rabczuk, Transfer learning enhanced physics informed neural network for phase-field modeling of fracture. *Theor. Appl. Fract. Mech.* **106**, 102447 (2020).

28. D. Z. Huang, K. Xu, C. Farhat, E. Darve, Learning constitutive relations from indirect observations using deep neural networks. *J. Comput. Phys.* **416**, 109491 (2020).

29. D. Elbrächter, P. Grohs, A. Jentzen, C. Schwab, Dnn expression rate analysis of high-dimensional PDEs: Application to option pricing. arXiv:1809.07669 (2018).

30. J. Han, A. Jentzen, W. E, Solving high-dimensional partial differential equations using deep learning. *Proc. Natl. Acad. Sci. U.S.A.* **115**, 8505–8510 (2018).

31. T. Poggio, H. Mhaskar, L. Rosasco, B. Miranda, Q. Liao, Why and when can deep-but not shallow-networks avoid the curse of dimensionality: A review. *Int. J. Autom. Comput.* **14**, 503–519 (2017).

32. Z. Li, N. Kovachki, K. Azizzadenesheli, B. Liu, K. Bhattacharya, A. Stuart, A. Anandkumar, Neural operator: Graph kernel network for partial differential equations. arXiv:2003.03485 (2020).

33. Z. Li, N. Kovachki, K. Azizzadenesheli, B. Liu, K. Bhattacharya, A. Stuart, A. Anandkumar, Multipole graph neural operator for parametric partial differential equations. arXiv:2006.09535 (2020).

34. Z. Li, N. Kovachki, K. Azizzadenesheli, B. Liu, K. Bhattacharya, A. Stuart, A. Anandkumar, Fourier neural operator for parametric partial differential equations. arXiv:2010.08895 (2020).

35. L. Lu, P. Jin, G. Pang, Z. Zhang, G. E. Karniadakis, Learning nonlinear operators via DeepONet based on the universal approximation theorem of operators. *Nat. Mach. Intell.* **3**, 218–229 (2021).

36. T. Chen, H. Chen, Universal approximation to nonlinear operators by neural networks with arbitrary activation functions and its application to dynamical systems. *IEEE Trans. Neural Netw.* **6**, 911–917 (1995).

37. A. D. Back, T. Chen, Universal approximation of multiple nonlinear operators by neural networks. *Neural Comput.* **14**, 2561–2566 (2002).

38. H. Kadri, E. Duflos, P. Preux, S. Canu, A. Rakotomamonjy, J. Audiffren, Operator-valued kernels for learning from functional response data. *J. Mach. Learn. Res.* **17**, 1–54 (2016).

39. M. Griebel, C. Rieger, Reproducing kernel hilbert spaces for parametric partial differential equations. *SIAM-ASA J. Uncertain. Quantif.* **5**, 111–137 (2017).

40. H. Owhadi, Do ideas have shape? plato's theory of forms as the continuous limit of artificial neural networks. arXiv:2008.03920 (2020).

41. N. H. Nelsen, A. M. Stuart, The random feature model for input-output maps between banach spaces. arXiv:2005.10224 (2020).

42. C. Schwab, J. Zech, Deep learning in high dimension: Neural network expression rates for generalized polynomial chaos expansions in uq. *Anal. Appl.* **17**, 19–55 (2019).

43. S. Wojtowytsch, W. E, Can shallow neural networks beat the curse of dimensionality? A mean field training perspective. *IEEE Trans. Artif. Intell.* **1**, 121–129 (2021).

44. S. Lanthaler, S. Mishra, G. E. Karniadakis, Error estimates for deeponets: A deep learning framework in infinite dimensions. arXiv:2102.09618 (2021).

45. S. Cai, Z. Wang, L. Lu, T. A. Zaki, G. E. Karniadakis, DeepM&Mnet: Inferring the electroconvection multiphysics fields based on operator approximation by neural networks. arXiv:2009.12935 (2020).

46. C. Lin, Z. Li, L. Lu, S. Cai, M. Maxey, G. E. Karniadakis, Operator learning for predicting multiscale bubble growth dynamics. arXiv:2012.12816 (2020).

47. B. Liu, N. Kovachki, Z. Li, K. Azizzadenesheli, A. Anandkumar, A. Stuart, K. Bhattacharya, A learning-based multiscale method and its application to inelastic impact problems. arXiv:2102.07256 (2021).

48. P. C. Di Leoni, L. Lu, C. Meneveau, G. Karniadakis, T. A. Zaki, Deeponet prediction of linear instability waves in high-speed boundary layers. arXiv:2105.08697 (2021).

49. Z. Mao, L. Lu, O. Marxen, T. A. Zaki, G. E. Karniadakis, DeepM&Mnet for hypersonics: Predicting the coupled flow and finite-rate chemistry behind a normal shock using neural-network approximation of operators. arXiv:2011.03349 (2020).

50. Y. Khoo, J. Lu, L. Ying, Solving parametric PDE problems with artificial neural networks. arXiv:1707.03351 (2017).

51. N. Geneva, N. Zabaras, Modeling the dynamics of PDE systems with physics-constrained deep auto-regressive networks. *J. Comput. Phys.* **403**, 109056 (2020).

52. Y. Chen, B. Dong, J. Xu, Meta-mgnet: Meta multigrid networks for solving parameterized partial differential equations. arXiv:2010.14088 (2020).

53. D. Kochkov, J. A. Smith, A. Alieva, Q. Wang, M. P. Brenner, S. Hoyer, Machine learning accelerated computational fluid dynamics. arXiv:2102.01010 (2021).

54. A. Griewank, On automatic differentiation, in *Mathematical Programming: Recent Developments and Applications* (Kluwer Academic Publishers, 1989), pp. 83–108.

55. A. G. Baydin, B. A. Pearlmutter, A. A. Radul, J. M. Siskind, Automatic differentiation in machine learning: A survey. *J. Mach. Learn. Res.* **18**, 1–43 (2018).

56. M. Tancik, P. P. Srinivasan, B. Mildenhall, S. Fridovich-Keil, N. Raghavan, U. Singhal, R. Ramamoorthi, J. T. Barron, R. Ng, Fourier features let networks learn high frequency functions in low dimensional domains. arXiv:2006.10739 (2020).

57. M. Raissi, H. Babaee, P. Givi, Deep learning of turbulent scalar mixing. *Phys. Rev. Fluids* **4**, 124501 (2019).

58. T. A. Driscoll, N. Hale, L. N. Trefethen, Chebfun guide (2014).

59. J. J. Park, P. Florence, J. Straub, R. Newcombe, S. Lovegrove, DeepSDF: Learning Continuous Signed Distance Functions for Shape Representation, *Proceedings of the IEEE/CVF Conference on Computer Vision and Pattern Recognition* (2019), pp. 165–174.

60. M. S. Selig, Uiuc airfoil data site (1996).

61. S. Wang, H. Wang, P. Perdikaris, On the eigenvector bias of fourier feature networks: From regression to solving multi-scale pdes with physics-informed neural networks. arXiv:2012.10047 (2020).

62. S. Wang, Y. Teng, P. Perdikaris, Understanding and mitigating gradient pathologies in physics-informed neural networks. arXiv:2001.04536 (2020).

63. S. Wang, X. Yu, P. Perdikaris, When and why PINNs fail to train: A neural tangent kernel perspective. arXiv:2007.14527 (2020).

64. L. McClenny, U. Braga-Neto, Self-adaptive physics-informed neural networks using a soft attention mechanism. arXiv:2009.04544 (2020).

65. J. Bradbury, R. Frostig, P. Hawkins, M. J. Johnson, C. Leary, D. Maclaurin, G. Necula, A. Paszke, J. VanderPlas, S. Wanderman-Milne, Q. Zhang, JAX: Composable transformations of Python+NumPy programs (2018).

66. J. D. Hunter, Matplotlib: A 2D graphics environment. *IEEE Ann. Hist. Comput.* **9**, 90–95 (2007).

67. C. R. Harris, K. J. Millman, S. J. van der Walt, R. Gommers, P. Virtanen, D. Cournapeau, E. Wieser, J. Taylor, S. Berg, N. J. Smith, R. Kern, M. Picus, S. Hoyer, M. H. van Kerkwijk, M. Brett, A. Haldane, J. F. del Río, M. Wiebe, P. Peterson, P. Gérard-Marchant, K. Sheppard, T. Reddy, W. Weckesser, H. Abbasi, C. Gohlke, T. E. Oliphant, Array programming with numpy. *Nature* **585**, 357–362 (2020).

68. C. Rasmussen, C. Williams, *Gaussian Processes for Machine Learning*, Adaptive Computation and Machine Learning (MIT Press, 2006).

69. D. P. Kingma, J. Ba, Adam: A method for stochastic optimization. arXiv:1412.6980 (2014).

70. C. Finn, P. Abbeel, S. Levine, *International Conference on Machine Learning* (PMLR, 2017), pp. 1126–1135.

71. A. Iserles, in *A First Course in the Numerical Analysis of Differential Equations* (Cambridge Univ. Press, 2009), no. 44.

72. E. Haghighat, M. Raissi, A. Moure, H. Gomez, R. Juanes, A physics-informed deep learning framework for inversion and surrogate modeling in solid mechanics. *Comput. Methods Appl. Mech. Eng.* **379**, 113741 (2021).

73. S. Wang, P. Perdikaris, Long-time integration of parametric evolution equations with physics-informed deeponets. arXiv:2106.05384 (2021).

74. S. M. Cox, P. C. Matthews, Exponential time differencing for stiff systems. *J. Comput. Phys.* **176**, 430–455 (2002).
